# Reovirus exerts potent oncolytic effects in head and neck cancer cell lines that are independent of signalling in the EGFR pathway

**DOI:** 10.1186/1471-2407-12-368

**Published:** 2012-08-24

**Authors:** Katie Twigger, Victoria Roulstone, Joan Kyula, Eleni M Karapanagiotou, Konstantinos N Syrigos, Richard Morgan, Christine White, Shreerang Bhide, Gerard Nuovo, Matt Coffey, Brad Thompson, Adel Jebar, Fiona Errington, Alan A Melcher, Richard G Vile, Hardev S Pandha, Kevin J Harrington

**Affiliations:** 1 Division of Cancer Biology Chester Beatty Laboratories, The Institute of Cancer Research, 237 Fulham Road, London, SW3 6JB, UK; 2 Department of Oncology, Sotiria General Hospital, Athens, Greece; 3 Postgraduate Medical School, The University of Surrey, Guildford, UK; 4 The Comprehensive Cancer Centre, Ohio State University, Columbus, Ohio, USA; 5 Oncolytics Biotech Inc, Calgary, Canada; 6 Leeds Institute of Molecular Medicine, Leeds, UK; 7 Molecular Medicine Program, Mayo Clinic, Rochester, MN, USA; 8 Targeted Therapy Laboratory, Division of Cancer Biology, Institute of Cancer Research, 237 Fulham Road, London, SW3 6JB, UK

**Keywords:** Biomarker, Cancer, EGFR, Ras, Reovirus, Oncolytic virus

## Abstract

**Background:**

Reovirus exploits aberrant signalling downstream of Ras to mediate tumor-specific oncolysis. Since ~90% squamous cell carcinomas of the head and neck (SCCHN) over-express EGFR and SCCHN cell lines are sensitive to oncolytic reovirus, we conducted a detailed analysis of the effects of reovirus in 15 head and neck cancer cell lines. Both pre- and post-entry events were studied in an attempt to define biomarkers predictive of sensitivity/resistance to reovirus. In particular, we analysed the role of EGFR/Ras signalling in determining virus-mediated cytotoxicity in SCCHN.

**Methods:**

To test whether EGFR pathway activity was predictive of increased sensitivity to reovirus, correlative analyses between reoviral IC50 by MTT assay and EGFR levels by western blot and FACS were conducted. Inhibition or stimulation of EGFR signalling were analysed for their effect on reoviral oncolysis by MTT assay, and viral growth by TCID50 assay. We next analysed the effects of inhibiting signalling downstream of Ras, by specific inhibitors of p38MAPK, PI3-K or MEK, on reoviral killing examined by MTT assay. The role of PKR in reoviral killing was also determined by blockade of PKR using 2-aminopurine and assaying for cell survival by MTT assay. The apoptotic response of SCCHN to reovirus was examined by western blot analysis of caspase 3 cleavage.

**Results:**

Correlative analyses between reoviral sensitivity and EGFR levels revealed no association. Intermediate sub-viral and core particles showed the same infectivity/cytotoxicity as intact reovirus. Therefore, sensitivity was not determined by cell entry. In 4 cell lines, oncolysis and viral growth were both unaffected by inhibition or stimulation of EGFR signalling. Inhibition of signalling downstream of Ras did not abrogate reoviral oncolysis and, in addition, modulation of PKR using 2-aminopurine did not alter reovirus sensitivity in resistant cell lines. Caspase 3 cleavage was not detected in infected cells and oncolysis was observed in pan-caspase inhibited cells.

**Conclusions:**

In summary, reovirus is potently oncolytic in a broad panel of SCCHN cell lines. Attempts to define sensitivity/resistance by analysis of the EGFR/Ras/MAPK pathway have failed to provide a clear predictive biomarker of response. Further analysis of material from *in vitro* and clinical studies is ongoing in an attempt to shed further light on this issue.

## Background

Reovirus is a small, non-enveloped double-stranded RNA virus, commonly isolated from the human respiratory or gastrointestinal tract [1]. Infection is widespread, with 50-100% of adults showing seropositivity [2,3]. However, reovirus is considered benign because most infections are either asymptomatic or result in only mild illness. Despite its lack of pathogenicity in humans, reovirus displays selective oncolytic activity against transformed and malignant cells [4,5]. Initial mechanistic studies showed that transfection with elements of the Ras signalling pathway, including EGFR and its constitutively active form v-erbB, sos and mutated Ras itself, increased the sensitivity of cells to reovirus-induced cell death [6,7]. The activated Ras signalling in these cells was subsequently found to inhibit the function of PKR, which in untransformed cells prevents viral protein translation. Thus, in Ras-activated cells, dysfunctional PKR signalling allows reovirus replication to proceed and cell death ensues [7,8].

Evidence from several studies into the precise molecular interactions linking increased Ras pathway activity and the regulation of reovirus oncolysis reveals a complex picture. NIH-3T3 fibroblast cells, transformed with activated forms of Ras, only supported reovirus replication if signalling from Ras to RalGEF/p38MAPK was intact [9]. When p38MAPK was inhibited in melanoma, reovirus-induced oncolysis was abrogated [10]. Together, this indicates that activity in the p38MAPK pathway is a determinant of sensitivity to reovirus in these cell types. Alternatively, in C26 colorectal tumour cells, reovirus-induced cell death was found to be distinct from Ras status and viral replication. In either the presence or absence of mutant Ras, C26 cells supported reovirus replication but expression of mutated Ras increased the sensitivity of tumour cells to reovirus-induced apoptosis [11]. Additional influences of Ras pathway status on the effects of reovirus infection have been highlighted by Marcato et al. (2007) who demonstrated significantly enhanced proteolytic disassembly (uncoating) of reovirus in Ras-transformed NIH-3T3 cells [12]. They also showed that Ras transformation increases the infectious:non-infectious particle ratio and promotes caspase-mediated release and spread of viral progeny.

In spite of differences in the reported mechanism of killing, preclinical studies in a wide range of *in vitro* and *in vivo* models, including intratumoural and intravenous injections in immune-deficient and -competent mice, have clearly shown that reovirus has a broad spectrum of oncolytic activity (reviewed in [13,14]). Clinical testing of reovirus through a strong translational programme is well advanced following phase I and II studies as a single agent [15-17] and in combination with cytotoxic chemotherapy [18-20] or radiotherapy [21]. Consequently, reovirus is currently being tested under a Special Protocol Agreement from the US Federal Drug Administration in a randomised phase III study of carboplatin and paclitaxel plus either placebo or reovirus in patients with relapsed/metastatic SCCHN ( http://clinicaltrials.gov/ct2/show/NCT01166542).

Overexpression of epidermal growth factor receptor (EGFR) and consequent activation of the Ras signalling pathway is the dominant oncogenic process in SCCHN [22]. Specific anti-EGFR monoclonal antibodies have already shown clinical benefits in newly diagnosed [23] and relapsed/metastatic SCCHN [24] and it is likely that novel agents that target the EGFR/Ras axis will be active in this disease. Therefore, we have conducted a detailed analysis of the effects of reovirus in a panel of head and neck cancer cell lines. Both pre- and post-entry events have been studied in an attempt to define biomarkers that will predict for sensitivity/resistance to reoviral therapy. In particular, we have analysed the role of the EGFR/Ras signalling pathway in determining virus-mediated cytotoxicity in SCCHN.

## Results

### Reovirus is active against a panel of head and neck cancer cell lines

**Figure 1:**
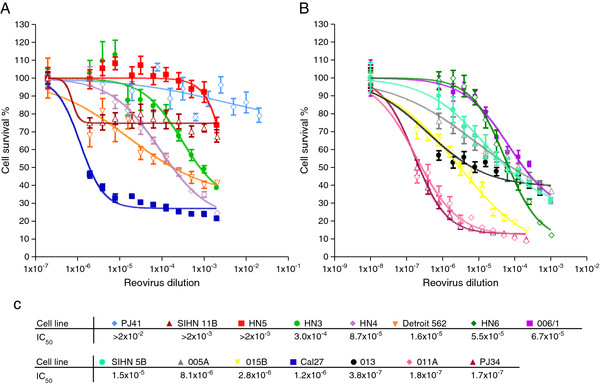
**HN cell lines have a wide range of reovirus IC**_**50**_** dilution values. A**, **B**. HN cells were infected with reovirus at 1.4×10^9^ TCID_50_/ml diluted 2 fold, starting from a 1:500, a 1:1000 or a 1:5000 dilution. Cell survival was assessed by MTT assay at 96 hours post-infection. Data were log transformed and plotted as sigmoidal dose response curves, with uninfected controls assigned an arbitrary value of 1×10^-8^. Means are from 3 independent experiments and error bars represent SEMs. **C**. IC_50_ dilutions of reovirus were interpolated from the dose response curves.

We initially sought to profile and define the sensitivity of human head and neck (SCCHN) tumour cells to reovirus-induced oncolysis. A panel of 15 previously characterised cell lines [25] were infected with serial dilutions of reovirus and assessed for cell survival. The SCCHN tumour cell lines showed a broad range of sensitivities to reovirus (Figure 1A, B). Using these data, the IC_50_ dilution of reovirus for each cell line was derived and the resulting values ranked (Figure 1C). HN3 and HN5 were chosen as examples of relatively resistant cell lines, with IC_50_ dilutions of 3.0 × 10^-4^ and >2 × 10^-3^, respectively, whereas Cal27 (1.2 × 10^-6^) and SIHN-5B (1.5 × 10^-6^) were selected as relatively sensitive to reovirus. These cell lines were used in many of the subsequent experiments in view of our previous experience of their reliable *in vitro* behaviour. 

### The method of reoviral entry into SCCHN cells does not predict their sensitivity

The main cellular receptor for reovirus is the junctional adhesion molecule-1 (JAM-1) [26]. Therefore, the level of JAM-1 expression was determined by FACS analysis on 4 representative cell lines with a spread of IC_50_ values of approximately 3 logs. JAM-1 expression was lowest in the most resistant cell line (HN5). However, HN5 cells still expressed measurable levels of JAM-1 and the highest level of receptor expression was seen in the second most resistant cell line (HN3). Overall, there was no clear evidence that the level of JAM-1 expression predicted for the variation in susceptibility to reovirus-induced cell death (Additional file 1).

Before reovirus can access the cytoplasm, capsid proteins, notably σ3 and μ1, are removed or altered by proteolysis [27,28]. This occurs either within endosomes or lysosomes following receptor binding and endocytosis of intact viral particles, or by extracellular digestion creating an intermediate or infectious subviral particle (ISVP), which can penetrate the membrane and enter the cytoplasm directly. Since Ras-transformed cells can secrete proteases, we investigated whether predigestion of reovirus particles could enhance their infectivity in SCCHN cells. In particular, we wished to test whether predigested reovirus would be more cytotoxic in the relatively resistant HN5 cell line.

**Figure 2:**
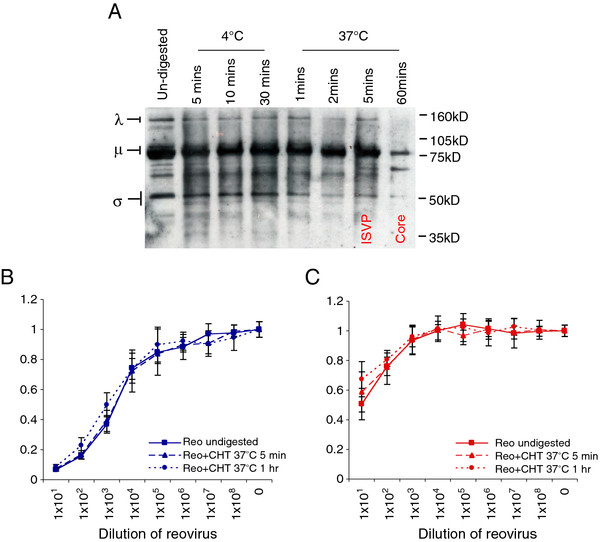
**Reovirus ISVPs and cores kill HN cells to the same extent as intact virus. A**. Reovirus stocks at 7.8×10^8^ TCID_50_/ml were treated with 10μg/ml chymotrypsin (CHT) for times and temperatures indicated. Viral proteins were resolved on 10% NuPage Novex Bis Tris gels and probed using reovirus goat anti-serum. **B**, **C**. Reovirus treated for 5 mins (ISVPs) or 1 hr (cores) at 37°C, was used to infect Cal27 (**B**) and HN5 (**C**) cells, alongside un-digested reovirus. Cell survival was assessed by MTT assay at 96 hours post-infection. Means are from 3 independent experiments and error bars represent SEMs.

Reovirus was treated at 37°C with chymotrypsin for either 5 mins to form ISVPs or 1 hour to give core particles. These digestion conditions were verified by the disappearance of λ, μ and σ proteins, detected by western blot (Figure 2A). Infection with ISVP and viral cores showed the same level of cytotoxicity in Cal27 cells as with undigested reovirus (Figure 2B). In HN5, the digested particles were impaired very slightly in their infectivity at the highest concentration at which they were exposed to the cells, but exhibited the same level of cell kill as untreated reovirus at all other dilutions (Figure 2C). These data demonstrate that generation of ISVPs through pre-entry proteolysis does not influence sensitivity to reovirus in SCCHN cells. Therefore, we next sought to investigate the intracellular interactions taking place during reovirus infection in this tumour type.

### Characterisation of EGFR expression in the SCCHN cell panel

**Figure 3:**
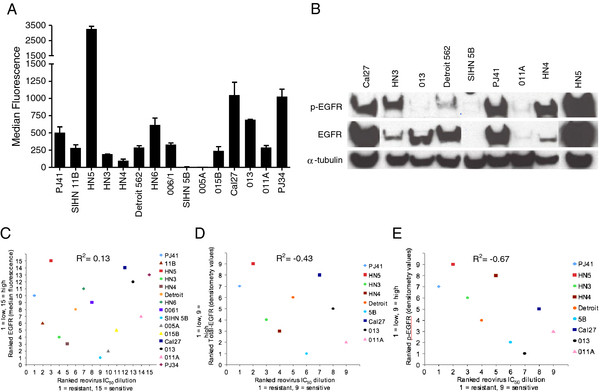
**HN cell lines show varying EGFR expression levels. A**. HN cells were stained with anti-EGFR primary antibody (7.5 μg/ml ICR62) then with 20 μg/ml FITC conjugated secondary antibody. Median fluorescence was determined by FACS analysis. Means are from at least 2 independent experiments and error bars represent SEMs. **B**. Lysates of HN cells indicated were analysed on 10% Precise Protein Gels and subsequently probed for total EGFR, phospho-Tyr1068 EGFR and α-tubulin for loading control. **C**, **D**, **E**. Reovirus IC_50_ dilutions were assigned rank values (1 = resistant, 15 = sensitive) and were correlated against ranked (**C**) median fluorescence levels, (**D**) ranked densitometry values for total EGFR or (**E**) phospho-tyr1068 EGFR (1 = EGFR low, 9 = EGFR high). R^2^ = 0.13, -0.43 and −0.67 respectively.

The dependence of reovirus oncolysis on upregulated Ras signalling has been reported previously [7,8]. Since Ras signalling can be driven by EGFR stimulation and SCCHN overexpresses EGFR, the panel of cell lines was evaluated for EGFR expression levels with a view to assessing whether reovirus sensitivity could be predicted by measuring EGFR expression. FACS analysis of EGFR expression was carried out for the whole panel and 9 representative cell lines were also profiled for total and phospho-EGFR by western blot. A broad range of cell surface EGFR levels was evident across the panel (Figure 3A). Similarly, total and phospho-EGFR protein levels were also widely distributed in the cell lines tested (Figure 3B). HN5 (median fluorescence value 3265) and Cal27 (median fluorescence value 1049) expressed the highest amounts of EGFR by FACS and western blot. Conversely, HN3 and SIHN-5B have relatively low levels of surface EGFR (median fluorescence 193 and 7, respectively). Levels of total and phospho-EGFR for SIHN-5B were undetectable by western blot, while HN3 had constitutively phosphorylated EGFR. Following profiling, the cell lines were ranked according to their EGFR expression by FACS and western analysis for either total or phospho-EGFR - resulting in 3 different ranks (Additional file 2: Table S1). To determine whether the FACS data correlate with total and/or phospho-EGFR, the ranked data were plotted against each other. FACS data vs total EGFR western blot showed a strong positive correlation (R^2^ = 0.90, P < 0.001) (Additional file 3). No correlation was evident between FACS analysis and phospho-EGFR western blot (R^2^ = 0.22, P = 0.58) (Additional file 4), revealing that surface level EGFR analysis represents the levels of total EGFR protein in each cell line, rather than the active signalling component. 

### Correlation between EGFR expression, GTP-loading on Ras and reovirus sensitivity

To test whether EGFR pathway activity, and, hence, signalling in the Ras pathway, was predictive of increased sensitivity to reovirus, the EGFR ranks obtained in Figure 3A and B were plotted against the ranks of reovirus IC_50_ dilution derived for the cell line panel (Figure 1C). Total EGFR assessed either by FACS or western blot did not correlate with reovirus IC_50_ dilution (Figure 3C, D (R^2^ = 0.13, P > 0.1 and −0.43, P > 0.1, respectively). Interestingly, a non-statistically significant inverse correlation was seen between phospho-EGFR and reovirus IC_50_ dilution (Figure 3E) (R^2^ = −0.67, P = 0.06).

**Figure 4:**
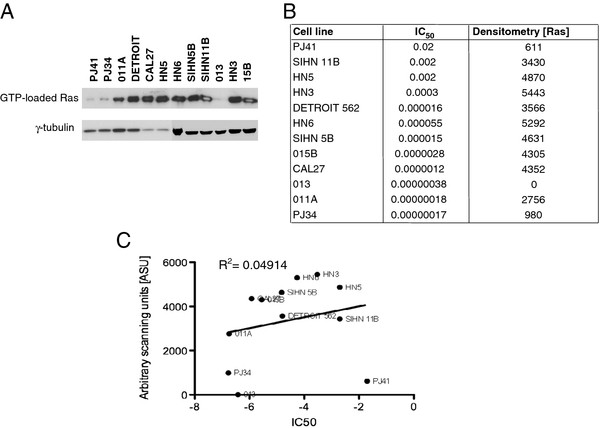
**GTP-loading status of Ras does not predict sensitivity of SCCHN cell lines to reovirus. A**. GTP-loading on Ras was determined for 12 cell lines in the SCCHN panel. **B**, **C**. Densitometry of GTP-loaded Ras (Densitometry [Ras]) does not correlate with reovirus IC50 values.

The baseline GTP-loading status of Ras was determined for 12 representative cell lines. The resulting western blot (Figure 4A) and densitometry data (Figure 4B) demonstrate that most cell lines had similar levels of Ras activation. Exceptions to this finding included SIHN 013, PJ41 and PJ34 cell lines. There was no significant correlation between Ras activation status and sensitivity to reovirus (as determined by IC_50_ dilution) (Figure 4C).

For further experiments, 4 cell lines were selected from the panel as being representative of the broad range of EGFR expression/reovirus sensitivity: HN5 (EGFR high, reovirus resistant); HN3 (EGFR low, reovirus resistant; Cal27 (EGFR high, reovirus sensitive); and SIHN 5B (EGFR low, reovirus sensitive).

### Epidermal Growth Factor Receptor stimulation or blockade does not affect reoviral cytotoxicity or growth

**Figure 5:**
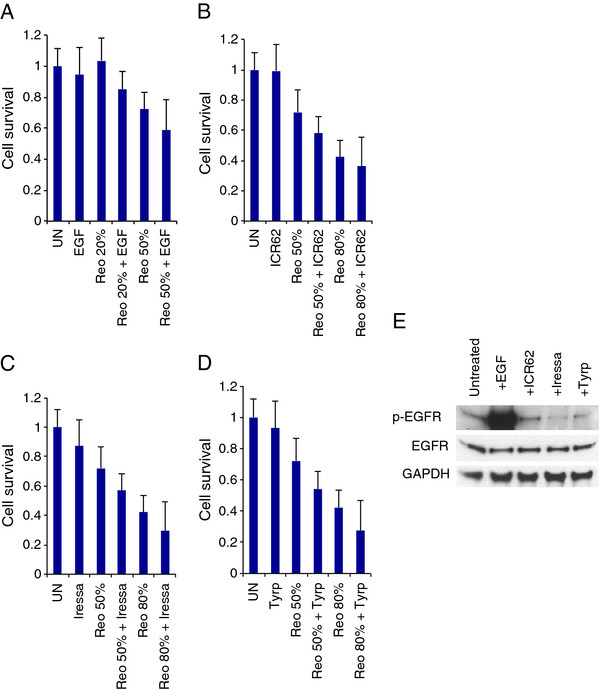
**Stimulation or inhibition of EGFR signalling does not affect reovirus cytotoxicity in Cal27 cells.** Cells were treated for 1 hr with 200 nM epidermal growth factor (EGF), 400nM anti-EGFR antibody (ICR62), 1 μM Iressa or 100 μM Tyrphostin AG99 (Tyrp), then either lysed, resolved on 8% Precise Protein Gels and probed for total EGFR, phospho-Tyr1068 EGFR and GAPDH or α-tubulin as loading controls, or infected with reovirus at 1.9×10^9^ TCID_50_/ml and assayed for cell survival by MTT at 96 hours post-infection. Reovirus was diluted as follows: 1:16000 (20%), 1:4000 (50%) and 1:500 (80%). **A**. EGF stimulation does not increase reoviral cytotoxicity. **B**, **C**, **D**. ICR62-, gefitinib- (Iressa) and Tyrphostin-mediated inhibition of EGFR did not inhibit reoviral cytotoxicity. Means are calculated from 3 independent experiments and error bars represent SEMs. **E**. Western blot analysis showing effect of EGF, ICR62, Gefitinib (Iressa) and Tyrphostin on EGFR signaling.

We next examined whether manipulation of EGFR signalling could change the sensitivity of the 4 selected cell lines to reovirus by stimulating or blocking the receptor, infecting cells with virus and measuring cell survival (Figure 5A-E, Additional file 5, Additional file 6, Additional file 7). Pre-treatment with EGF did not alter cell survival post-reovirus infection in all 4 cell lines (P > 0.1), although treatment with EGF alone was markedly cytotoxic to HN5 (Additional file 7). Blockade of the receptor using an anti-EGFR antibody to inhibit ligand binding (ICR62) [29] or using tyrosine kinase inhibitors to inactivate the signalling capability of the receptor (Iressa/Gefitinib or Tyrphostin-AG99) also had no effect on cell survival following infection with reovirus (P > 0.1 for all analyses). The activity of the EGFR inhibitors was tested in the context of stimulation by EGF (Additional file 8). Both ICR62 and Iressa/Gefitinib effectively inhibited phosphorylation of EGFR, but Tyrphostin-AG99 was inactive. HN5 exhibited a previously documented sensitivity to Iressa/Gefitinib [30]. 

**Figure 6:**
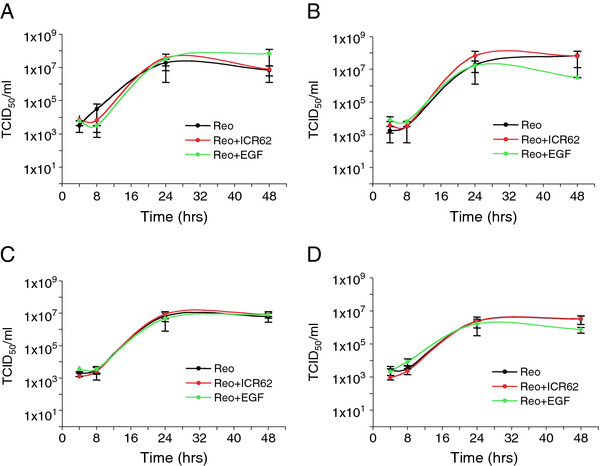
**Reovirus grows at the same rate in EGFR-stimulated or -inhibited SCCHN cells as in untreated cells. A**. Cal27, **B**. SIHN 5B, **C**. HN3 and **D**. HN5, were treated overnight with 200 nM epidermal growth factor (EGF) or 400 nM anti-EGFR antibody (ICR62) then infected with reovirus (MOI 10) using viral stocks at 1.9×10^9^ TCID_50_/ml. Ligands were replaced 2 hrs post infection. Cells and supernatants were harvested at the times indicated for TCID_50_ titration on L929 cells. Means are from at least 2 independent experiments and error bars represent SEMs.

It has been reported that activated Ras signalling blocks the anti-viral action of PKR and permits increased reoviral replication [7,8]. Therefore, we tested the effect of EGFR stimulation and inhibition on reoviral growth. Cells were pre-incubated with EGF, ICR62 or media alone and then infected with reovirus. At various time points after infection the cells and their supernatants were harvested and titred by TCID_50_ assay. Neither stimulation by EGF nor inhibition by ICR62 affected the growth of reovirus in the 4 cell lines tested (Figure 6A-D). This result was further confirmed using gefitinib/iressa (Additional file 9). Interestingly, all 4 of the cell lines showed the same level of reoviral replication, despite their differing susceptibility to reovirus-induced cell death indicating that high or low replication rates do not account for the range of reovirus sensitivities observed (Additional file 10). 

### Reovirus cytotoxicity does not depend on PI3-K, MAPK or p38MAPK signalling

**Figure 7:**
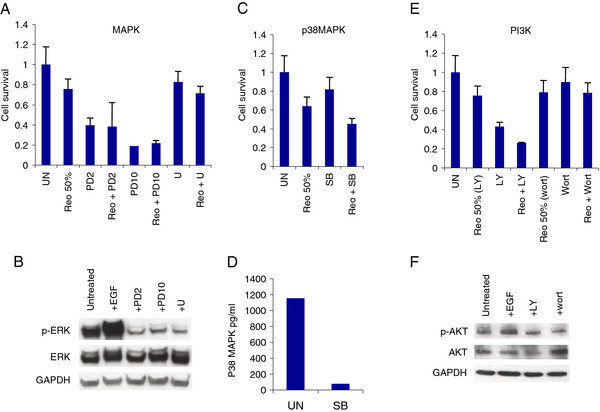
**MEK, PI3-K or p38MAPK inhibition does not affect reovirus cytotoxicity.** Cal27 cells were inhibited for 2 hrs with 2 μM (PD2) or 10 μM (PD10) PD184352, 10 μM U0126 (U), 10 μM SB202190 (SB), 10 μM LY294003 (LY) or 1 μM wortmannin (wort). Monolayers were then either lysed, resolved on 8% Precise Protein Gels (MAPK) or 10% NuPage Novex Bis Tris gels (PI3-K) and probed for total ERK1/2, phosho-Thr202 ERK1/2, total AKT, phospho-Ser473 AKT and GAPDH or β-actin as loading controls, or infected with reovirus at 1.2×10^10^ TCID_50_/ml (PD, SB and LY) or 7.8×10^8^ TCID_50_/ml (wort) and assayed for cell survival by MTT. p38MAPK target knock-down was confirmed by ELISA. Reovirus was diluted at 1:4000 for 50% cell kill. **A**, **B**. MAPK inhibition. **C**, **D**. p38MAPK inhibition. **E**, **F**. PI3K inhibition. Means are calculated from at least 3 independent experiments and error bars represent SEMs.

Having examined the influence of EGFR itself on reoviral oncolysis in SCCHN, we went on to determine whether inhibition of downstream signalling effectors could influence sensitivity to reovirus. We targeted the three major signalling pathways downstream of Ras - MAPK, PI3-K and p38MAPK. To inhibit MEK in the MAPK pathway, the specific tyrosine kinase inhibitors PD184352 (PD) and U0126 (U) were used [31,32]. PD was employed at 2 different concentrations, 2 μM to target MEK1/2 only and 10 μM for blockade of MEK1/2 and MEK5. LY294002 (LY) and wortmannin (wort) were utilised to block PI3-K [33], and p38MAPK was inhibited by SB202190 (SB) [34]. Following incubation with inhibitors, cells were infected with reovirus and cell survival was analysed. Inhibitor activity was confirmed by western analysis for all pathways except p38MAPK, where the many isoforms of p38MAPK makes this type of analysis unsuitable. Instead, we confirmed p38MAPK blockade by SB by means of ELISA. Reoviral cytotoxicity in SCCHN was not abrogated by blockade in any of the 3 pathways tested, with cell survival being equal to or less than reovirus infection alone (Figure 7A-F, Additional file 11, Additional file 12, Additional file 13) (P > 0.05 for all analyses). For p38MAPK inhibition by SB, in all cell lines the agent had little single agent cytotoxic activity and did not reduce reovirus-induced cell kill. For PI3K inhibition, LY induced significant cell death in 3 of the 4 cell lines (Figure 7, Additional file 11 and 12) but this was not the case with wortmannin. Again, there was no evidence that either LY or wortmannin was capable of abrogating the cytotoxicity of reovirus. 

However, for the analyses involving PD184352, it was clear that this agent exerted significant single agent activity at both 2 and 10 μM concentration and this raised concerns that this effect might have masked an inhibitory effect on reovirus cytotoxicity. Thus, we decided to subject the combination of reovirus and PD184352 to formal combination index analysis according to the methodology of Chou and Talalay [35]. Initially, we defined IC_50_ values for PD184352 in SIHN-5B, Cal27, HN3 and HN5 cells (data not shown) and then combined fixed ratios (0.5, 1.0 and 2.0 IC_50_) of the IC_50_ of reovirus and PD184352 and analysed cell survival by MTT assay as described previously. These data demonstrated striking synergy between reovirus and MEK inhibition for all cell lines (Additional file 14).

Therefore, taken together, these data suggest that unlike earlier observations made in transformed fibroblasts, reoviral cytotoxicity is not dependent on the activation of downstream effectors of Ras in SCCHN. In fact, reovirus appears to show a surprising synergistic interaction with MEK inhibition across all 4 cell lines tested when the agents are combined at ratios close to the IC_50_.

### Pharmacological inhibition of PKR phosphorylation does not restore reovirus sensitivity to resistant cells

**Figure 8:**
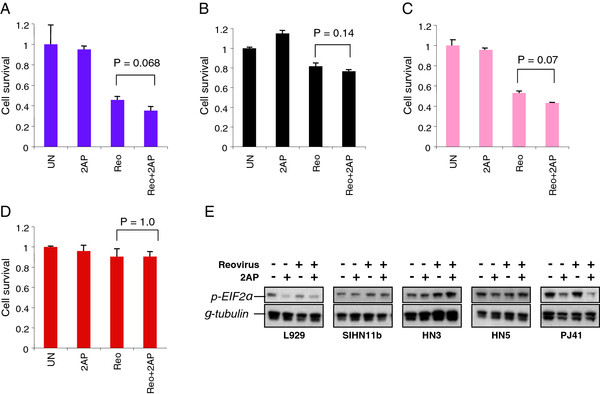
**Inhibition of PKR does not augment reovirus cytotoxicity in HN cells. A**. SIHN 11B, **B**. PJ41, **C**. HN3, **D**. HN5, were incubated with the PKR inhibitor 2-aminopurine (2AP) for 1hr prior to infection with reovirus (MOI 50) using viral stocks at 7.8×10^8^ TCID_50_/ml. Cell survival was assessed by MTT assay at 96 hours post-infection. Data are representative of 3 independent experiments and error bars are SEMs. **E**. L929, HN3, HN5, PJ41 and SIHN11b were plated at 1×10^6^/10 cm dish and treated the following day with 2AP (1 mM). After 1 hour incubation with 2AP, reovirus was added at an MOI 50. Cells were incubated for a further 4 hours and then harvested for western analysis with p-EIF2α and γ-tubulin for loading controls.

Transformation of reovirus resistant fibroblasts with intermediates of the EGFR and Ras signalling pathway was previously shown to inactivate PKR and, thereby, allow viral protein synthesis to proceed [7]. To determine the role of PKR in reoviral killing in SCCHN, 4 relatively reovirus-resistant cell lines were incubated with 2-AP then infected and assayed for cell survival. Although the presence of 2-AP marginally increased cytotoxicity in 3 of the cell lines, the effect did not reach statistical significance (SIHN-11B (Figure 8A, P = 0.068), PJ41 (Figure 8B, P = 0.14), HN3 (Figure 8C, P = 0.07) or HN5 (Figure 8D, P = 1.0)). These data suggest that the oncolytic effect of reovirus in these cells is not controlled by PKR inactivation. 2-AP had no effect on reoviral cytotoxicity in the sensitive Cal27 cell line (data not shown). 

Given the fact that these findings do not mirror previously reported findings in transformed NIH-3T3 cells [7], we analysed the effect of reovirus infection and 2-AP treatment on L929 cells and the 4 relatively reovirus-resistant head and neck cancer cell lines using immunocytochemistry to measure p-PKR staining and western analysis to define downstream phosphorylation of EIF2α (which acts to inhibit viral protein translation). In L929 cells, reovirus infection had little effect on p-PKR staining or p-EIF2α protein levels, although 2-AP reduced both of these signals in the absence or presence of reovirus infection, confirming activity of the drug (Additional file 15, Figure 8E). Similarly, both at the level of immunocytochemistry (Additional file 15) and (more variably) on western analysis (Figure 8E), 2-AP was shown to reduce the p-PKR and p-EIF2α signal as a single agent therapy, confirming drug-on-target effect for this agent. Interestingly, in 3 of the 4 head and neck cancer cell lines, reovirus infection increased p-PKR staining (Additional file 15) and this was not reversible with 2-AP. p-EIF2α remained unchanged or increased in response to reovirus infection in all 4 head and neck cancer cell lines and was only reduced by 2-AP in PJ41 cells (although this did not correlate with increased reovirus cytotoxicity). In fact, the western analysis data from PJ41 cells more closely resembled those from L929 cells. Taken together, these data demonstrate that although 2-AP is biologically active in uninfected reovirus-resistant head and neck cancer cell lines, it does not prevent reovirus-induced phosphorylation of PKR and downstream phosphorylation of p-EIF2α and does not increase reovirus-induced cytotoxicity.

### Interferon signalling does not predict reovirus sensitivity

In view of the fact that many viruses trigger innate immune activation, the profile of interferon secretion before and after reovirus infection was determined in Cal27, HN3, HN5 and SIHN-5B cells by ELISA assay for interferon-α, -β and γ Additional file 16). In the uninfected state, there was no clear correlative pattern between reovirus sensitivity and baseline interferon secretion, which was limited to interferon-β. For example, the most resistant cell line (HN5) had unmeasurable basal secretion of interferon-α, -β and γ whereas the next most resistant cell line (HN3) secreted the highest levels of interferon-β. In response to reovirus infection, interferon secretion (mainly interferon-β) was increased in Cal27, HN5 and SIHN-5B cell lines, but the pattern did not correlate with sensitivity to reovirus. Thus, although the lowest level of interferon-β signalling was seen in the most sensitive cell line (Cal27), the highest level of interferon-α and –β signalling was seen in the next most sensitive cell line (SIHN-5B).

### Reovirus-induced cell death is not apoptotic in SCCHN

**Figure 9:**
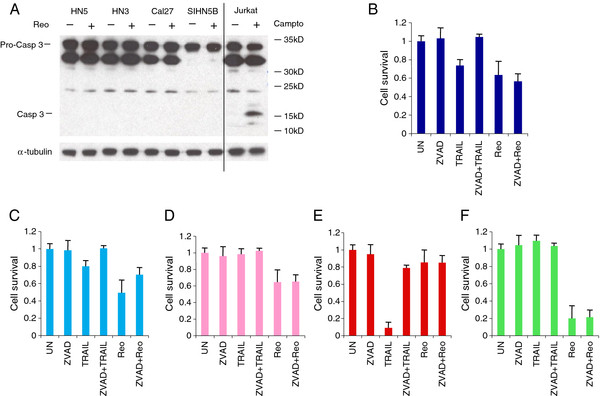
**Reovirus induced cell death is not apoptotic in SCCHN cells. A**. SCCHN cells shown were infected with reovirus (MOI 1) using viral stocks at 3.7×10^9^ TCID_50_/ml and Jurkat cells were treated with 10 μM camptothecin (campto). Lysates were resolved on 10% NuPage Novex Bis Tris gels and probed for pro-caspase 3 cleavage. **B**. Cal27, **C**. SIHN 5B, **D**. HN3, **E**. HN5 and **F**. 011A were treated with pan-caspase inhibitor (ZVAD, 200 μM) for 2 hr prior to either TNFα-related apoptosis inducing ligand treatment (TRAIL 100ng/ml) or reovirus infection (MOI 10) using viral stocks at 1.9 × 10^9^ TCID_50_/ml. Cell survival was assessed by MTT assay at 96 hours post-infection. Means are from at least 3 independent experiments and error bars represent SEMs.

Previous reports have suggested that for some cells the effect of Ras activation on reoviral cytotoxicity might be mediated by sensitising the cells to virally-induced apoptosis, rather than determining their ability to support viral replication. Our finding that both resistant and sensitive SCCHN cells support reovirus replication to the same extent (Additional file 10) raises the possibility that this effect may also be operating in SCCHN. Therefore, the apoptotic response of SCCHN to reovirus was examined by western blot analysis of caspase 3 cleavage. Jurkat cells treated with 10 μM camptothecin were used as a positive control and showed the 19kDa caspase 3 cleavage product (Figure 9A). In contrast, reovirus did not induce apoptosis in the 4 SCCHN cell lines tested (Figure 9A).

This result was confirmed by incubating SCCHN cells with the pancaspase inhibitor z-VAD-FMK (ZVAD), prior to reovirus infection or treatment with the exogenous apoptosis-inducing ligand TRAIL (as a positive control), and measuring cell survival. Varying responses to reovirus and TRAIL were observed in the different cell lines. Specifically, TRAIL treatment was associated with reduced cell survival in 3 of the 5 SCCHN cell lines tested and in all cases ZVAD was effective in partially reversing cytotoxicity. TRAIL reduced cell survival in Cal27 and this was inhibited by ZVAD treatment. However, the level of reovirus-induced cell kill was similar in the presence or absence of ZVAD (P > 0.1) (Figure 9B). Pre-incubation of SIHN 5B with ZVAD also abrogated TRAIL-induced cytotoxicity, but reovirus oncolysis was also non-statistically significantly inhibited by ZVAD treatment (P = 0.1) in these cells (Figure 9C). HN3 cells were resistant to the effects of TRAIL and reovirus-induced cell kill was unaffected by the presence of ZVAD (P > 0.1) (Figure 9D). HN5 cells were extremely sensitive to TRAIL-induced apoptosis, which was almost fully reversed by treatment with ZVAD. However, as observed above, this cell line was largely resistant to reovirus and this was not altered by ZVAD treatment (P > 0.1) (Figure 9E). In contrast, 011A were completely insensitive to TRAIL and highly sensitive to reovirus-induced cell death, but this was not affected by pre-incubation with ZVAD (P > 0.1) (Figure 9F). Taken together, these results indicate that reovirus-induced cell death in SCCHN cells does not involve caspase 3 activation and is not inhibited by pancaspase blockade. Therefore, in marked contrast to melanoma cell lines [36], reovirus killing of SCCHN cells appears to be non-apoptotic.

## Discussion

The translational development of reovirus has progressed at a rapid rate through a series of phase I and II clinical trials that have been driven by an active programme of preclinical research (reviewed in [37]). Reovirus has been shown to be active against a wide variety of tumour types and to mediate synergistic therapeutic interactions with either chemotherapy [18,19,38,39] or radiotherapy [21,40]. As a result of this work, reovirus is currently being tested in combination with carboplatin-paclitaxel doublet chemotherapy in a phase III study in patients with platin-refractory SCCHN.

The initial studies on the mechanism of reovirus-induced killing of tumour cells suggested that Ras pathway activation (by oncogenic mutation or upstream dysregulation) was a key determinant of viral replication and subsequent oncolysis [6,7]. This raised the prospect of using Ras mutation or pathway activation status as a biomarker to guide patient selection for reovirus therapy in clinical studies. However, further mechanistic studies have shown that the situation is highly complex and, as yet, no definitive biomarker of sensitivity to reovirus has been defined. Therefore, in most ongoing studies of oncolytic reovirus, the state of activation of the EGFR/Ras axis is not used as an entry requirement or as a stratification factor. Since SCCHN has emerged as an extremely important clinical target for oncolytic reovirus therapy, we undertook a detailed analysis of the factors that might predict sensitivity to treatment in SCCHN with a view to defining predictive biomarkers for testing in future clinical studies. In particular, our initial hypothesis was that the sensitivity of SCCHN to reovirus would largely depend on the signalling status in the EGFR/Ras/MAPK axis.

In initial studies, we profiled the relative sensitivities of a panel of 15 SCCHN cell lines and saw a 5-log range in IC_50_ (Figure 2A, B). Subsequent analysis of EGFR expression by flow cytometry (in 15 cell lines) and by western analysis for total and phospho-EGFR (in 9 cell lines) showed significant variability between individual SCCHN cell lines (Figure 3A, B), but no statistically significant correlations with reovirus sensitivity (Figure 3C-E). Indeed, if anything, there was a trend towards a negative correlation between phospho-EGFR on western analysis and reovirus sensitivity by IC_50_ estimation (Figure 3E). Further studies in which we quantitated GTP-loading on Ras (Figure 4) and modulated signalling through the EGFR/Ras/MAPK axis (Figures 5, 6, 7, Additional file 5, Additional file 6, Additional file 7, Additional file 9, Additional file 11, Additional file 12, Additional file 13) failed to provide a clear indication of a cellular marker of sensitivity or resistance to reovirus. Indeed, it is interesting to note that the extent of *in vitro* reoviral replication did not correlate with cytotoxicity in SCCHN cells. In this respect, the data are similar to those obtained in other studies using C26 colorectal tumour cells but, in direct contrast to those findings, the mechanism of death in SCCHN cells was non-apoptotic (Figure 9).

Therefore, despite clear evidence that there can be significant variability (> 5 logs) in the susceptibility of SCCHN to reovirus-induced cytotoxicity, detailed profiling of pre- and post-entry events has failed to define a clear signaling biomarker (or combination of biomarkers) of sensitivity or resistance. These findings have a number of implications. Most importantly, it is clear that, at least at the present time, an attempt to select SCCHN patients for oncolytic reovirus therapy on the basis of putative biomarkers in the EGFR/Ras/MAPK pathway is not a viable strategy. In regard to the ongoing phase III study in patients with relapsed/metastatic head and neck cancers, our data provide reassurance that the eligibility criteria that allow entry of patients with platin-refractory disease, irrespective of EGFR/Ras/MAPK pathway status, are appropriate. We cannot exclude the possibility that EGFR/Ras signaling may ultimately have some significant predictive value for reovirus therapy in SCCHN, especially in the light of the extensive interconnectivity and redundancy of signaling pathways within tumour cells. This would be consistent with findings in other oncolytic systems in which early indications of specific genetic dependencies for oncolytic specificity turned out to be more complex than initially thought [41]. In addition, our studies here focus exclusively upon the genetic determinants of reovirus replication in tumour cells in culture. It is well established that sensitivity to viral replication and cytolysis *in vitro* can sometimes bear little relation to *in vivo* sensitivity of a tumour type, especially in the context of immunocompetent models [42]. We profiled innate immune response (at baseline and in response to viral infection) in 4 representative SCCHN cell lines and saw no clear correlation with reovirus sensitivity (Additional file 16). However, the screens that we have performed here do not take into account the dependence of innate immune responses to viral infection, in both tumor cells and host immune effectors, upon cell signaling pathways, such as EGFR/Ras, in the tumour cells. Therefore, it is possible that many components of the complex relations between sensitivity to reovirus infection, replication, cytolysis and tumor therapy remain to be elucidated.

Looking forwards, our *in vitro* findings provide a strong rationale for collecting tumour samples from the patients currently enrolling in clinical protocols as a driver for further biomarker discovery studies. It will be especially useful to obtain pre- and post-treatment samples from the large number of patients entering the reovirus clinical programme and to correlate findings from genomic, transcriptomic and proteomic studies on tumour and normal tissues with the clinical outcome data. Indeed, we are currently adopting this approach across a broad panel of tumour cell types in *in vitro* analyses to provide guidance for the use of precious patient samples obtained in ongoing and future clinical studies with reovirus.

In the setting of SCCHN, it is also useful to interpret our data in the context of similar attempts to define biomarkers for treatment response to anti-EGFR-targeted monoclonal antibodies, such as cetuximab/erbitux, zalutumumab and panitumumab [43]. Despite our ability to design chimeric, humanised or fully human antibodies with exquisite selectivity for a precisely designed target (EGFR) and the clear demonstration that these agents mediate a therapeutic effect in SCCHN, we are apparently no closer to defining biomarkers to predict which patients with this disease will and will not respond to anti-EGFR monoclonal antibody targeted therapy. This fact most likely highlights both the complexity of interplay between elements of the downstream signalling pathways and the limitations of trying to fully define the pathway by studying one element at a time. If this is true for a relatively simple biologic such as a monoclonal antibody, perhaps we should not be surprised that the same is true for a complex, multi-faceted agent like an oncolytic virus.

## Conclusions

In summary, we have shown that reovirus is potently oncolytic in a broad panel of SCCHN cell lines. Attempts to define sensitivity/resistance by analysis of the EGFR/Ras/MAPK pathway have failed to provide a clear predictive biomarker. Further analysis of material from *in vitro* and clinical studies is ongoing in an attempt to shed further light on this issue.

## Methods

### Cells

Detroit-562, Cal27, 006/1, 005A, 013, HN3, HN4, HN5, HN6, 015B, SIHN-5B, 011A, SIHN-11B, (head and neck cancer) were cultured in Dulbecco’s Modified Eagle’s Medium (DMEM). PJ41 and PJ34 (head and neck cancer) were cultured in Iscove’s Modified Eagle’s Medium (IMEM, Gibco, Invitrogen, Paisley, UK) and Jurkat (leukemia) were cultured in Roswell Park Memorial Institute media (RPMI). DMEM and IMEM were supplemented with 5% (v/v) FCS and RPMI with 10% (v/v) FCS (PAA, Pasching, Austria). All media contained 1% (v/v) L-glutamine and 0.5% (v/v) penicillin/streptomycin and cells were kept at 37°C in a humidified atmosphere containing 10% CO_2_. All cell lines were obtained from Dr S Eccles, ICR, UK, except for Jurkat, which was obtained from Prof. R. Marais, ICR, UK.

### Oncolytic Reovirus

Reovirus (Dearing Type 3) was obtained from Oncolytics^TM^ Biotech Inc. (Calgary, Canada) and stored at −80°C. Neat stocks were in phosphate-buffered Saline (PBS) and 1:10 working dilutions were stored in DMEM containing 2% (v/v) FCS, 1% (v/v) glutamine and 0.5% (v/v) penicillin/streptomycin (plating media). New stocks of working dilutions were made periodically and titred by standard Tissue Culture Infectious Dose 50 (TCID_50_) assay on L929 cells, as described previously [39].

### Reagents

Recombinant human EGF (R&D Systems, Abingdon, UK), along with the EGFR inhibitors Iressa/Gefitinib (Biaffin, Kassel, Germany) Tyrphostin-AG99 (Calbiochem, Merck Chemicals, Nottingham, UK) and EGFR blocking antibody ICR62 (from Dr Sue Eccles, ICR, UK) were used in cell kill assays, western blot and one-step growth curve assays. MEK1/2 inhibitor U0126, PI3K inhibitor LY294002, p38 MAPK inhibitor SB202190 MEK1/2 and MEK 5 inhibitor PD184352 (all from Calbiochem, Merck Chemicals, Nottingham, UK) and Wortmannin (Sigma-Aldrich, Gillingham, UK) were used in cell kill and western blot analyses. ZVAD (R&D Systems, Abingdon, UK), chymotrypsin (CHT, Roche Applied Science, Burgess Hill, UK) and 2-aminopurine (2AP, Sigma Aldrich, Gillingham, UK) were used in cell kill assays. Camptothecin, (Sigma-Aldrich, Gillingham, UK) was used as a positive control for the induction of apoptosis in western blots.

### Cell survival experiments

Cells were seeded at 5x10^3^ in 96-well plates and incubated at 37°C for 24hrs before experimental conditions were applied. Where used, cells were treated with antibodies, inhibitors and ligands for 1–2 hrs before infection. Additional plating media was added to the wells 2-24hrs after infection and cell survival was assessed 96 hrs post-infection by MTT assay as described previously [10]. Reovirus IC_50_ values were determined by interpolation from a sigmoidal dose response curve fit of the log transformed survival data, derived using GraphPad Prism version 4.0c for Mac OS X (GraphPad Software Inc. San Diego, USA).

### ISVPs and cores

Reovirus stocks were treated with a final concentration of 10μg/ml sequencing grade CHT reconstituted in 1 mM HCl plus sequencing buffer, as per manufacturer’s instructions. Following digestion the CHT was neutralised with FCS and equal volumes of virus were analysed by western blot (see below). Proteins were detected using polyclonal anti-reovirus goat serum (Oncolytics Biotech Inc). Rabbit anti-goat HRP-conjugated antibody (Pierce, Perbio, Aalst, Belgium) was used for secondary detection. For cell kill analyses ISVP and core particles were created as above, diluted out in plating media and used to infect cells. Survival was analysed as described above.

### Assessment of cell surface EGFR

Cells were cultured in T175 flasks, harvested and 1×10^6^ cells stained with ICR62 for 1 hr at 4°C. Primary antibody binding was detected using F(ab’)2 rabbit anti-rat FITC conjugated IgG (Serotec, Oxford, UK). Staining was analysed using a FACSCalibur machine (Becton Dickinson, Oxford, UK).

### Western blots

Cells were incubated at 37°C for 24hrs before treatment with inhibitors. Monolayers were washed twice with PBS and scraped into 200 μl of lysis buffer (LB), supplemented with complete mini protease inhibitor cocktail tablets (Roche Applied Science, Gillingham, UK) for EGFR and ERK1/2 detection, phosphatase and protease inhibitors, as previously described for AKT analysis [10], and 10 μg/ml TLCK, 1 mM PMSF and a 1:100 dilution of protease cocktail I (all Sigma-Aldrich, Gillingham, UK) for pro-caspase 3 assay. Lysates were loaded into pre-cast sodium dodecyl sulfate-polyacrylamide (SDS-PAGE) gels, either Precise Protein gels (Pierce, Perbio, Aalst, Belgium) or NuPage Novex Bis-Tris gels (Invitrogen, Paisley, UK). Following electrophoresis proteins were transferred to polyvinylidene fluoride (PVDF) membranes and probed with specific primary antibodies as follows: murine anti-EGFR (Sigma-Aldrich, Gillingham, UK), rabbit anti-pY1068 EGFR (Invitrogen, Paisley, UK) rabbit anti-p44/42 MAPK, rabbit anti-phospsho-p44/42 MAPK, rabbit anti-AKT, rabbit anti-phospho-AKT, rabbit anti-EIF2α, rabbit anti-caspase 3 (all Cell Signalling Technology, Danvers, USA). Incubations with primary antibodies were followed by secondary labelling using sheep anti-mouse HRP (Amersham Biosciences, GE Healthcare, Amersham, UK) or goat anti-rabbit (Santa Cruz Biotechnology, Santa-Cruz, USA). SuperSignal West Pico Chemiluminescent Substrate (Pierce, Perbio, Aalst, Belgium) was used according to the manufacturers instructions for detection. Membranes were stripped between antibody staining procedures in Restore Western Blot Stripping Buffer (Pierce, Perbio, Aalst, Belgium) for 15mins at 37°C. Murine anti-α tubulin or anti-α tubulin (Sigma-Aldrich, Gillingham, UK), murine anti-GAPDH (Novus Biologicals, Littleton, USA) or rabbit anti-β actin (Cell Signalling Technology, Danvers, USA) were used for loading controls.

### Active Ras Pull-Down and Detection

Cells were grown so they were sub-confluent in T75 flasks prior to harvesting, processing and western blotting for Ras small GTPase activation using the Active Ras Pull-Down and Detection Kit (Thermo Scientific, Rockford, IL, USA). Experiments were performed per protocol according to the manufacturer’s instructions.

### One-step viral growth assays

Cells were seeded at 1×10^5^ in 24 well plates and treated o/n at 37°C with plating media alone, or plating media containing EGFR ligand/inhibitors. The following day cells were infected with reovirus for 2 hrs. Monolayers were washed once with PBS and the ligand/inhibitors replaced. Cells were scraped into the supernatant and harvested at time points post-infection, freeze-thawed three times and titred by TCID_50_ assay on L929 cells, as described previously [39].

### p38MAPK ELISA

Cells were plated at 5 × 10^5^ in 6 cm dishes. Cells were treated with SB202190 (10 μM) for 2 hours, harvested, and analysed for phospho-p38 (Surveyor IC: Human/Mouse/Rat Phospho-p38∝ (T180/Y182) immunoassay #SUV869, R&D Systems, Minneapolis, USA). Experiments were performed according to the protocol provided for the assay by the manufacturers.

### Interferon ELISA

Cal27, HN3, HN5 and SIHN-5B cells plated at 1 × 10^6^ in 10 cm dishes were treated with reovirus at an MOI of 5, or left untreated. Cells were incubated for 24 hours and supernatants were collected and spun down to remove cell debris. Samples were stored at −20°C until analysis for alpha, beta and gamma interferon by ELISA. IFN-α was analysed using match-paired antibodies from Mabtech, IFN-γ with match-paired antibodies from BD Biosciences and IFN-β using a kit from PBL Interferon Source according to the manufacturer’s instructions. Data were read on a Multiskan EX plate reader (Thermo Scientific) at 405 nm using Ascent software.

### JAM-1 FACS Analysis

Cells were harvested with trypsin, pelleted and resuspended in FACS buffer (1% FCS in PBS). 1 × 10^5^ cells in 100 μL were stained with 2 μL of JAM-A antibody (1H2A9, Santa Cruz, USA) or isotype control (mouse IgG2b-PE, Santa Cruz) and incubated for 30 minutes at 4°C. One millilitre of FACS buffer was added and cells were pelleted. Pellets were either resuspended in 500 μL PBS and analysed within an hour using a FACSCalibur machine (Becton Dickinson, Oxford, UK), or fixed in 1% paraformaldehyde for analysis within 5 days.

### Statistics

The data on EGFR status and reovirus cell killing were not normally distributed. Therefore Spearman’s rank correlation was used to test the correlation between EGFR status and reovirus cytotoxicity. A non-parametric test (Wilcoxon signed rank test) was used for testing of significance when evaluating the effects of agonists and inhibitors of the RGFR/Ras pathway on reovirus-induced cytotoxicity.

## Competing interests

Matt Coffey is a shareholder and employee of Oncolytics Biotech Inc., and Gerard Nuovo, Richard Vile, Alan Melcher and Kevin Harrington received funding from Oncolytics Biotech Inc in support of laboratory research.

## Authors’ contributions

KT, VR, JK and EMK were responsible for the planning, conducting and analysis of experimental work. KNS, AAM, RGV, HSP and KJH were responsible for planning and designing experiments and analysing data. SB, KT and VR were responsible for the statistical analysis, GN for the analysis by confocal microscopy and RM and CLW conducted experiments. MC and BT founded Reolysin® (Oncolytics Biotech, Inc) and were involved in experimental design of the study. AJ and FE conducted and analysed the interferon ELISA experiments, and KJH and VR were involved in writing the manuscript. All authors read and approved the final manuscript.

## Pre-publication history

The pre-publication history for this paper can be accessed here:

http://www.biomedcentral.com/1471-2407/12/368/prepub

## Supplementary Material

Additional file 1 **Junctional Adhesion Molecule-1 (JAM1) expression is similar in cell lines with widely differing IC50 values for reovirus.** A. HN5, B. HN3, C. Cal27, D. SIHN-5B. Mean fluorescence intensity values are indicated and are representative of at least 3 repeat experiments. Click here for file

Additional file 2 **Table S1.** Cell lines ranked according to their EGFR expression by FACS and western analysis for either total or phospho-EGFR. Click here for file

Additional file 3 **EGFR ranked (1 = EGFR low, 9 = EGFR high) by FACS (median fluorescence levels) and western blot (densitometry) correlate (R**^2^** = 0.90).** Click here for file

Additional file 4 **EGFR ranked by median fluorescence levels on FACS (1 = EGFR low, 9 = EGFR high) and by densitometry for phospho-tyr1068 EGFR western blot (1 = pEGFR low, 9 = pEGFR high) do not correlate (R**^2^** = 0.22).** Click here for file

Additional file 5 **Stimulation or inhibition of EGFR signalling does not affect reovirus cytotoxicity in SIHN-5B cells.** Cells were treated for 1 hr with 200nM epidermal growth factor (EGF), 400nM anti-EGFR antibody (ICR62), 1μM Iressa or 100μM Tyrphostin AG99 (Tyrp), then either lysed, resolved on 8% Precise Protein Gels and probed for total EGFR, phospho-Tyr1068 EGFR and GAPDH or α-tubulin as loading controls, or infected with reovirus at 1.9×10^9^ TCID_50_/ml and assayed for cell survival by MTT. Reovirus was diluted as follows: 1:64000 (20%) 1:8000 (50%) and 1:500 (80%). **A**. EGF stimulation does not increase reoviral cytotoxicity. **B**, **C**, **D**. ICR62-, gefitinib- (Iressa) and Tyrphostin-mediated inhibition of EGFR did not inhibit reoviral cytotoxicity. Means are calculated from 3 independent experiments and error bars represent SEMs. **E**. Western blot analysis showing effect of EGF, ICR62, Gefitinib (Iressa) and Tyrphostin on EGFR signaling. Click here for file

Additional file 6 **Stimulation or inhibition of EGFR signalling does not affect reovirus cytotoxicity in HN3 cells.** Cells were treated for 1 hr with 200nM epidermal growth factor (EGF), 400nM anti-EGFR antibody (ICR62), 1μM Iressa or 100μM Tyrphostin AG99 (Tyrp), then either lysed, resolved on 8% Precise Protein Gels and probed for total EGFR, phospho-Tyr1068 EGFR and GAPDH or α-tubulin as loading controls, or infected with reovirus at 1.9×10^9^ TCID_50_/ml and assayed for cell survival by MTT at 96 hours post-infection. Reovirus was diluted as follows: 1:32000 (20%) 1:2000 (50%) and 1:100 (80%). **A**. EGF stimulation does not increase reoviral cytotoxicity. **B**, **C**, **D**. ICR62-, gefitinib- (Iressa) and Tyrphostin-mediated inhibition of EGFR did not inhibit reoviral cytotoxicity. Means are calculated from 3 independent experiments and error bars represent SEMs. **E**. Western blot analysis showing effect of EGF, ICR62, Gefitinib (Iressa) and Tyrphostin on EGFR signaling. Click here for file

Additional file 7 **Stimulation or inhibition of EGFR signalling does not affect reovirus cytotoxicity in HN5 cells.** Cells were treated for 1 hr with 200nM epidermal growth factor (EGF), 400 nM anti-EGFR antibody (ICR62), 1 μM Iressa or 100 μM Tyrphostin AG99 (Tyrp), then either lysed, resolved on 8% Precise Protein Gels and probed for total EGFR, phospho-Tyr1068 EGFR and GAPDH or α-tubulin as loading controls, or infected with reovirus at 1.9×10^9^ TCID_50_/ml and assayed for cell survival by MTT at 96 hours post-infection. Reovirus was diluted as follows: 1:200 (20%) 1:100 (50%) and 1:50 (80%). **A**. EGF stimulation does not increase reoviral cytotoxicity. **B**, **C**, **D**. ICR62-, gefitinib- (Iressa) and Tyrphostin-mediated inhibition of EGFR did not inhibit reoviral cytotoxicity. Means are calculated from 3 independent experiments and error bars represent SEMs. **E**. Western blot analysis showing effect of EGF, ICR62, Gefitinib (Iressa) and Tyrphostin on EGFR signaling. Click here for file

Additional file 8 **The EGFR inhibitors ICR62 and Iressa are active in the context of stimulation by EGF.** Cells were treated with 400nM ICR62, 5mM Iressa or 10uM Tryphostin for 2 hours prior to treatment with 200nM EGF. Cell were then harvested an hour later for analysis of EGFR by western blot. Click here for file

Additional file 9 **Reovirus grows at the same rate in EGFR inhibited SCCHN cells as in untreated cells.** (A) Cal27, (B) SIHN 5B, (C) HN3 and (D) HN5, were treated overnight with 1 μM Iressa then infected with reovirus (MOI 10) using viral stocks at 1.2×10^10^ TCID_50_/ml. Iressa was replaced 2 hrs post infection. Cells and supernatants were harvested at the times indicated for TCID_50_ titration on L929 cells. Means are from at least 2 independent experiments and error bars represent SEMs. Click here for file

Additional file 10 **Reovirus grows at the same rate in reovirus sensitive or resistant cells. Cal27, SIHN 5B HN3 and HN5 infected with reovirus (MOI 10) using viral stocks at 1.9×10**^9^**TCID**_50_**/ml.** Cells and supernatants were harvested at the times indicated for TCID_50_ titration on L929 cells. Means are from at least 2 independent experiments and error bars represent SEMs. Click here for file

Additional file 11 **MEK, PI3-K or p38MAPK inhibition does not affect reovirus cytotoxicity in SIHN-5B cells.** Cells were inhibited for 2 hrs with 2 μM (PD2) or 10 μM (PD10) PD184352, 10 μM U0126 (U), 10 μM SB202190 (SB), 10 μM LY294003 (LY) or 1 μM wortmannin (wort). Monolayers were then either lysed, resolved on 8% Precise Protein Gels (MAPK) or 10% NuPage Novex Bis Tris gels (PI3-K) and probed for total ERK1/2, phosho-Thr202 ERK1/2, total AKT, phospho-Ser473 AKT and GAPDH or β-actin as loading controls, or infected with reovirus at 1.2×10^10^ TCID_50_/ml (PD, SB and LY) or 7.8×10^8^ TCID_50_/ml (wort) and assayed for cell survival by MTT. p38MAPK target knock-down was confirmed by ELISA. Reovirus was diluted at 1:6000 for 50% cell kill. **A**, **B**. MAPK inhibition. **C**, **D**. p38MAPK inhibition. **E**, **F**. PI3K inhibition. Means are calculated from at least 3 independent experiments and error bars represent SEMs. Click here for file

Additional file 12 **MEK, PI3-K or p38MAPK inhibition does not affect reovirus cytotoxicity in HN3 cells.** Cells were inhibited for 2 hrs with 2 μM (PD2) or 10 μM (PD10) PD184352, 10 μM U0126 (U), 10 μM SB202190 (SB), 10 μM LY294003 (LY) or 1 μM wortmannin (wort). Monolayers were then either lysed, resolved on 8% Precise Protein Gels (MAPK) or 10% NuPage Novex Bis Tris gels (PI3-K) and probed for total ERK1/2, phosho-Thr202 ERK1/2, total AKT, phospho-Ser473 AKT and GAPDH or β-actin as loading controls, or infected with reovirus at 1.2×10^10^ TCID_50_/ml (PD, SB and LY) or 7.8×10^8^ TCID_50_/ml (wort) and assayed for cell survival by MTT at 96 hours post-infection. p38MAPK target knock-down was confirmed by ELISA. Reovirus was diluted at 1:2000 for 50% cell kill. **A**, **B**. MAPK inhibition. **C**, **D**. p38MAPK inhibition. **E**, **F**. PI3K inhibition. Means are calculated from at least 3 independent experiments and error bars represent SEMs. Click here for file

Additional file 13 **MEK, PI3-K or p38MAPK inhibition does not affect reovirus cytotoxicity in HN5 cells.** Cells were inhibited for 2 hrs with 2 μM (PD2) or 10 μM (PD10) PD184352, 10 μM U0126 (U), 10 μM SB202190 (SB), 10 μM LY294003 (LY) or 1 μM wortmannin (wort). Monolayers were then either lysed, resolved on 8% Precise Protein Gels (MAPK) or 10% NuPage Novex Bis Tris gels (PI3-K) and probed for total ERK1/2, phosho-Thr202 ERK1/2, total AKT, phospho-Ser473 AKT and GAPDH or β-actin as loading controls, or infected with reovirus at 1.2×10^10^ TCID_50_/ml (PD, SB and LY) or 7.8×10^8^ TCID_50_/ml (wort) and assayed for cell survival by MTT at 96 hours post-infection. p38MAPK target knock-down was confirmed by ELISA. Reovirus was diluted at 1:100 for 50% cell kill. **A**, **B**. MAPK inhibition. **C**, **D**. p38MAPK inhibition. **E**, **F**. PI3K inhibition. Means are calculated from at least 3 independent experiments and error bars represent SEMs. Click here for file

Additional file 14 **Reovirus in combination with PD184352 is synergistic.** Cal27, HN3, HN5 SIHN5b were plated at 5×10^3^/ 96 well dish and treated the following day with 200 μl of 2x, 1x, 0.5x calculated IC_50_ doses of reovirus, PD184352, or the agents in combination. Cells were analysed for cell survival 96 hours later by MTT assay at 96 hours post-infection. Data are derived from 2 independent experiments ± SEM. **A**. Interaction between the treatment combinations was assessed using the method of Chou and Talalay [34]. Combination index (CI) values were calculated where CI <0.9 is classed as synergy, 0.9-1.1 is additive and >1.1 is antagonistic. **B**. Representative CI values for the combination of reovirus with PD184352 for Cal27, HN3, HN5 and SIHN5b. Click here for file

Additional file 15 **L929, SIHN11b, HN3, HN5 and PJ41 cells were seeded at 1 × 10**^6^**in 10 cm dishes, treated the following day with reovirus and were fixed with 10% formalin at room temperature for 4–8 hours then harvested and centrifuged at 1200 rpm for 5 minutes.** The remaining pellet was resuspended in sterile DEPC water before analysis by immunocytochemistry staining for p-PKR (by G. Nuovo). (PDF 137 kb) Click here for file

Additional file 16 **Interferon response does not predict reovirus sensitivity.** Cal27, HN3, HN5 SIHN5b were plated at 1×10^6^/10 cm dish and the following day treated with reovirus at an MOI of 5 (reo), or left untreated (Un). Cells were incubated for 24 hours and the the supernatants were harvested, spun down at 1200 rpm for 4 minutes to remove cells and debris, and the supernatant transferred to a fresh tube and stored at −20°C. Samples were analysed in triplicate for interferons by ELISA. Click here for file
