# Supplementary figures and images for: Reovirus exerts potent oncolytic effects in head and neck cancer cell lines that are independent of signalling in the EGFR pathway
Source: BMC Cancer. 2012 Aug 24;12:368. doi: 10.1186/1471-2407-12-368 (PMC3537694; doi:10.1186/1471-2407-12-368)

ADDITIONAL FILE 1.

**A**

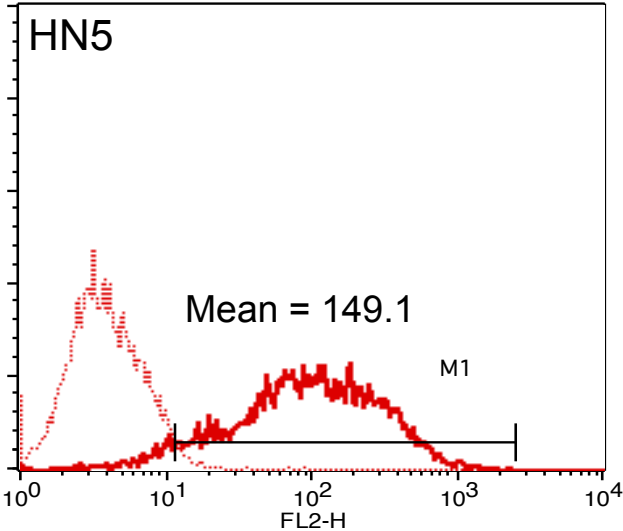

**B**

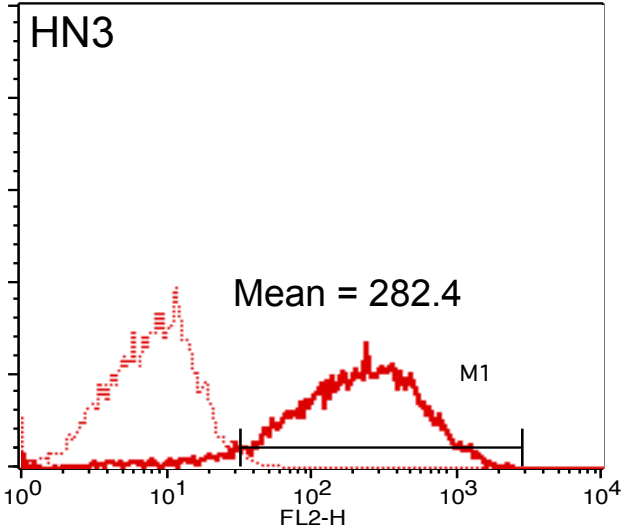

**C**

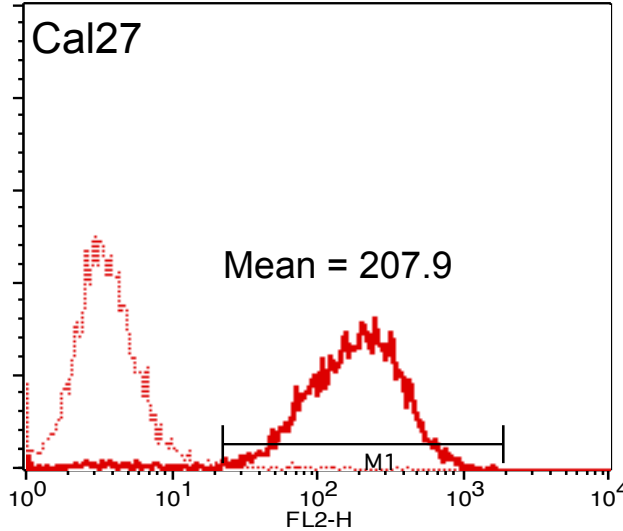

**D**

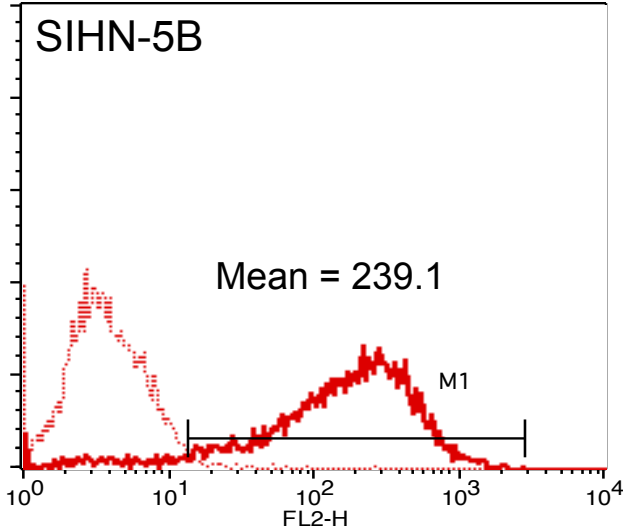

Supplement: Additional file 1 — Junctional Adhesion Molecule-1 (JAM1) expression is similar in cell lines with widely differing IC50 values for reovirus. A. HN5, B. HN3, C. Cal27, D. SIHN-5B. Mean fluorescence intensity values are indicated and are representative of at least 3 repeat experiments. [file 1471-2407-12-368-S1.pdf]

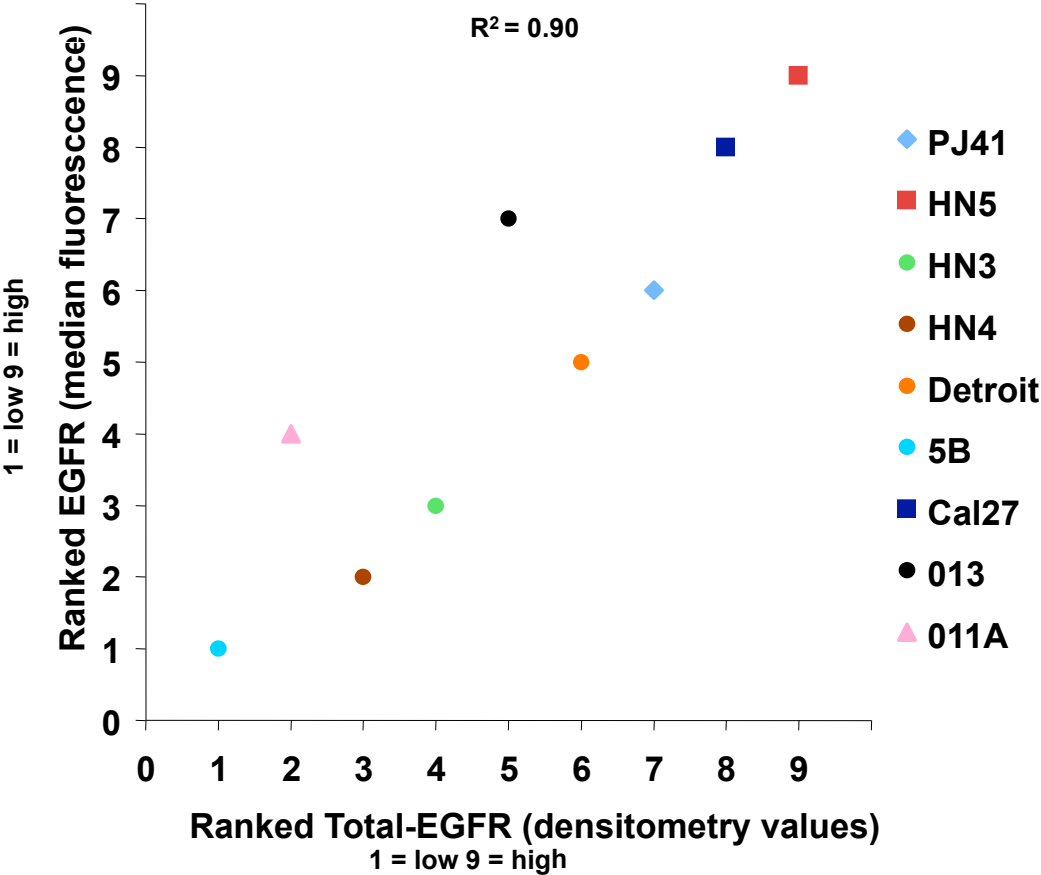

Supplement: Additional file 3 — EGFR ranked (1 = EGFR low, 9 = EGFR high) by FACS (median fluorescence levels) and western blot (densitometry) correlate (R2 = 0.90). [file 1471-2407-12-368-S3.pdf]

ADDITIONAL FILE 3.

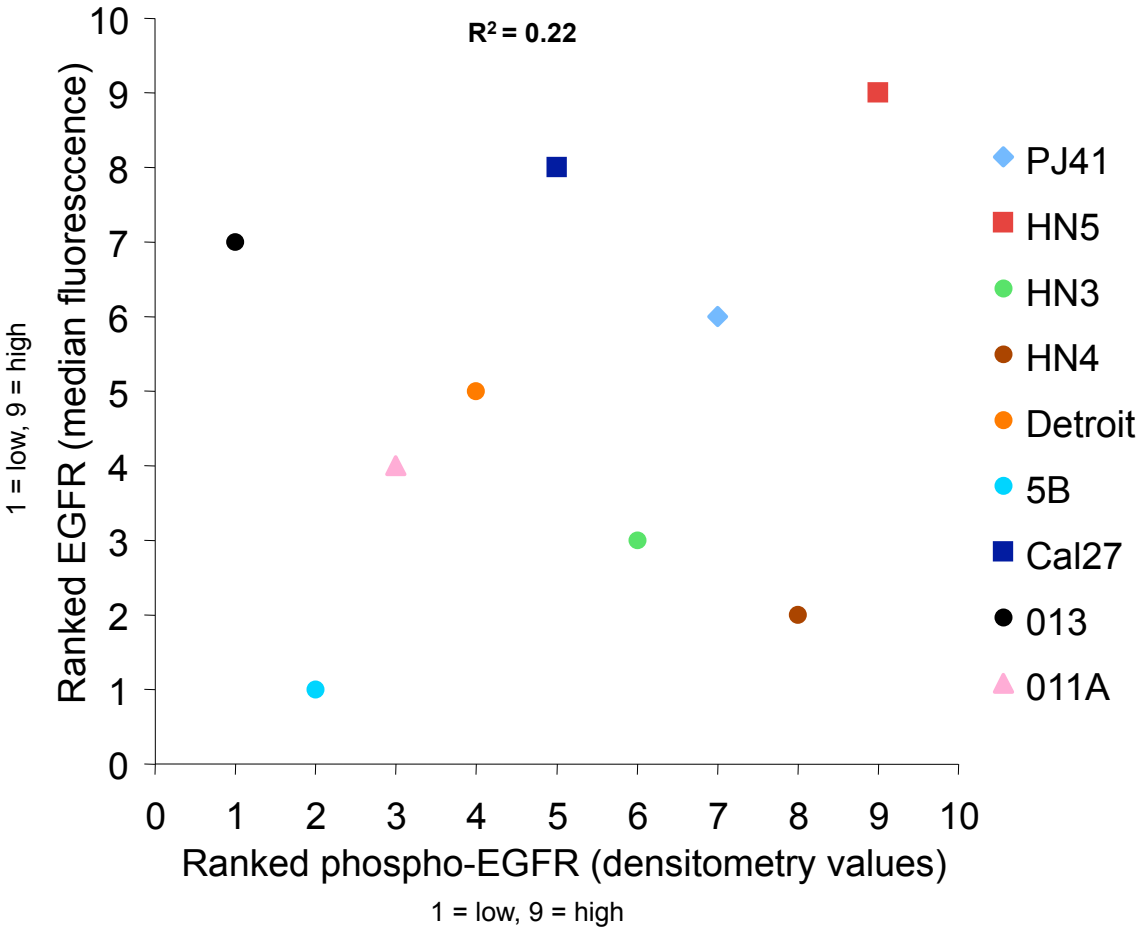

Supplement: Additional file 4 — EGFR ranked by median fluorescence levels on FACS (1 = EGFR low, 9 = EGFR high) and by densitometry for phospho-tyr1068 EGFR western blot (1 = pEGFR low, 9 = pEGFR high) do not correlate (R2 = 0.22). [file 1471-2407-12-368-S4.pdf]

ADDITIONAL FILE 4.

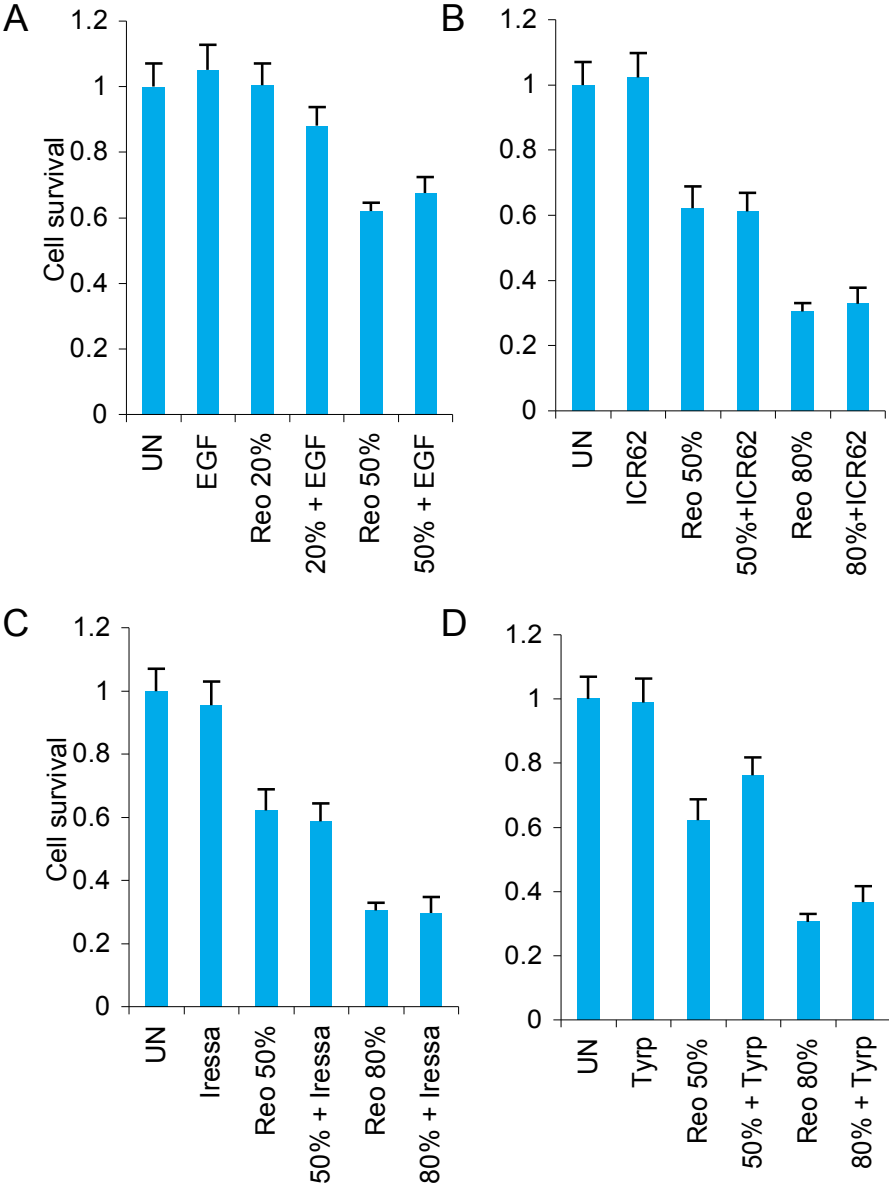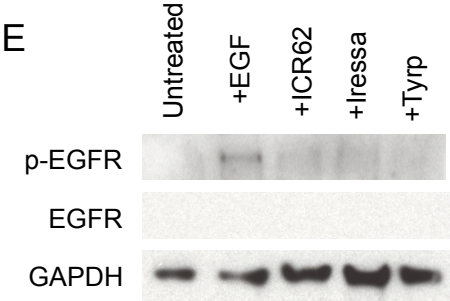

Supplement: Additional file 5 — Stimulation or inhibition of EGFR signalling does not affect reovirus cytotoxicity in SIHN-5B cells. Cells were treated for 1 hr with 200nM epidermal growth factor (EGF), 400nM anti-EGFR antibody (ICR62), 1μM Iressa or 100μM Tyrphostin AG99 (Tyrp), then either lysed, resolved on 8% Precise Protein Gels and probed for total EGFR, phospho-Tyr1068 EGFR and GAPDH or α-tubulin as loading controls, or infected with reovirus at 1.9×10^9^ TCID50/ml and assayed for cell survival by MTT. Reovirus was diluted as follows: 1:64000 (20%) 1:8000 (50%) and 1:500 (80%). A. EGF stimulation does not increase reoviral cytotoxicity. B, C, D. ICR62-, gefitinib- (Iressa) and Tyrphostin-mediated inhibition of EGFR did not inhibit reoviral cytotoxicity. Means are calculated from 3 independent experiments and error bars represent SEMs. E. Western blot analysis showing effect of EGF, ICR62, Gefitinib (Iressa) and Tyrphostin on EGFR signaling. [file 1471-2407-12-368-S5.pdf]

ADDITIONAL FILE 5.

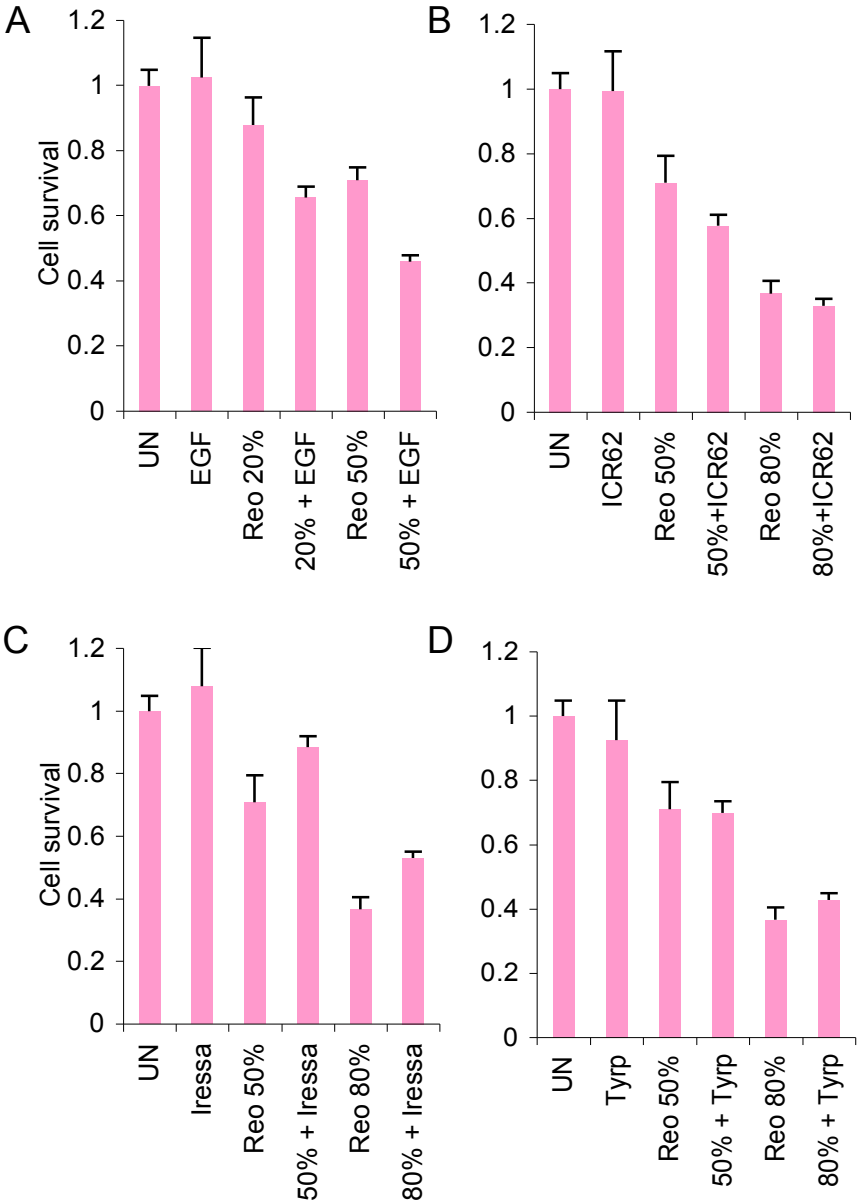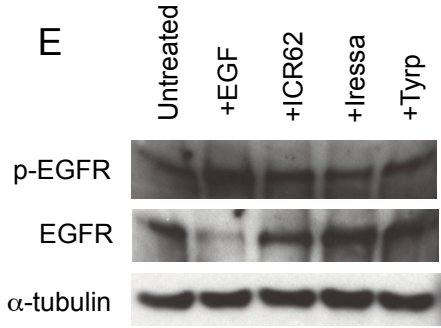

Supplement: Additional file 6 — Stimulation or inhibition of EGFR signalling does not affect reovirus cytotoxicity in HN3 cells. Cells were treated for 1 hr with 200nM epidermal growth factor (EGF), 400nM anti-EGFR antibody (ICR62), 1μM Iressa or 100μM Tyrphostin AG99 (Tyrp), then either lysed, resolved on 8% Precise Protein Gels and probed for total EGFR, phospho-Tyr1068 EGFR and GAPDH or α-tubulin as loading controls, or infected with reovirus at 1.9×10^9^ TCID50/ml and assayed for cell survival by MTT at 96 hours post-infection. Reovirus was diluted as follows: 1:32000 (20%) 1:2000 (50%) and 1:100 (80%). A. EGF stimulation does not increase reoviral cytotoxicity. B, C, D. ICR62-, gefitinib- (Iressa) and Tyrphostin-mediated inhibition of EGFR did not inhibit reoviral cytotoxicity. Means are calculated from 3 independent experiments and error bars represent SEMs. E. Western blot analysis showing effect of EGF, ICR62, Gefitinib (Iressa) and Tyrphostin on EGFR signaling. [file 1471-2407-12-368-S6.pdf]

ADDITIONAL FILE 6.

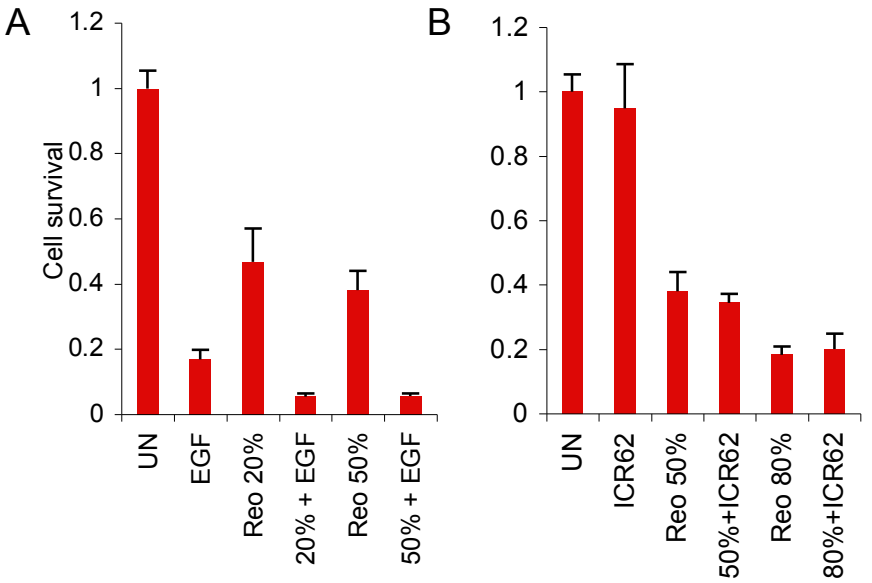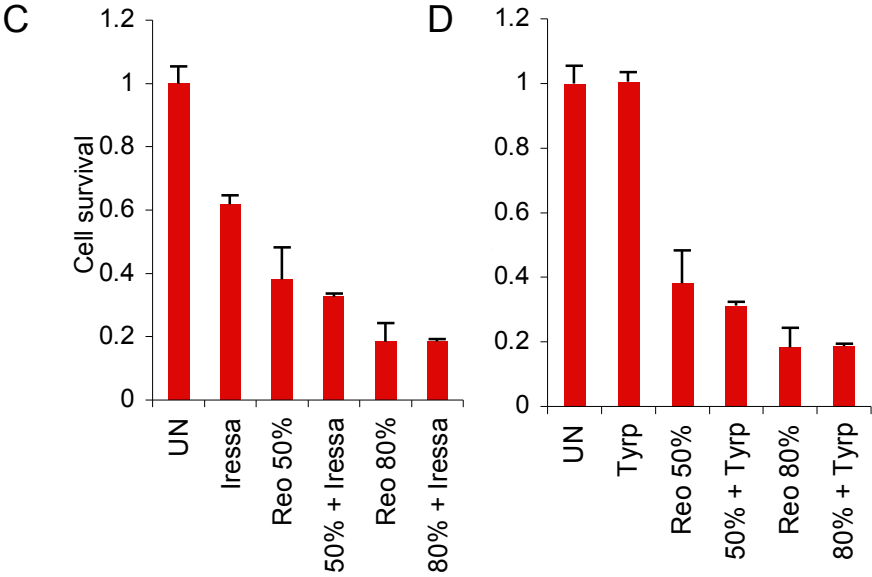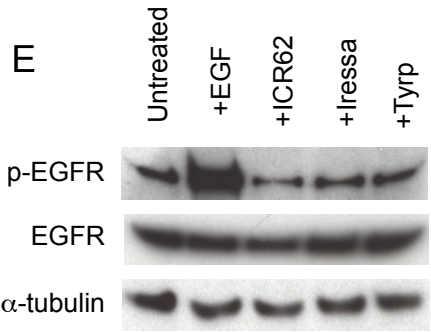

Supplement: Additional file 7 — Stimulation or inhibition of EGFR signalling does not affect reovirus cytotoxicity in HN5 cells. Cells were treated for 1 hr with 200nM epidermal growth factor (EGF), 400 nM anti-EGFR antibody (ICR62), 1 μM Iressa or 100 μM Tyrphostin AG99 (Tyrp), then either lysed, resolved on 8% Precise Protein Gels and probed for total EGFR, phospho-Tyr1068 EGFR and GAPDH or α-tubulin as loading controls, or infected with reovirus at 1.9×10^9^ TCID50/ml and assayed for cell survival by MTT at 96 hours post-infection. Reovirus was diluted as follows: 1:200 (20%) 1:100 (50%) and 1:50 (80%). A. EGF stimulation does not increase reoviral cytotoxicity. B, C, D. ICR62-, gefitinib- (Iressa) and Tyrphostin-mediated inhibition of EGFR did not inhibit reoviral cytotoxicity. Means are calculated from 3 independent experiments and error bars represent SEMs. E. Western blot analysis showing effect of EGF, ICR62, Gefitinib (Iressa) and Tyrphostin on EGFR signaling. [file 1471-2407-12-368-S7.pdf]

ADDITIONAL FILE 7.

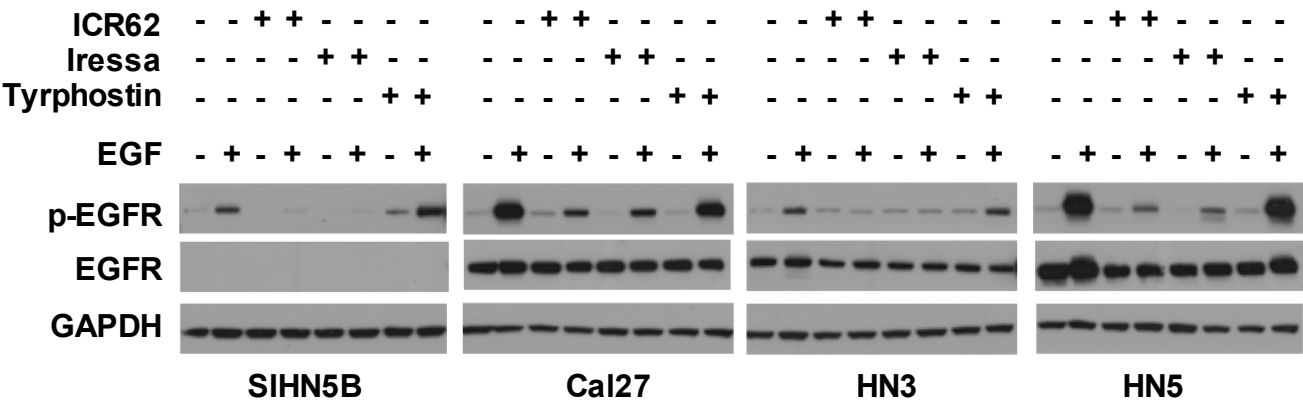

Supplement: Additional file 8 — The EGFR inhibitors ICR62 and Iressa are active in the context of stimulation by EGF. Cells were treated with 400nM ICR62, 5mM Iressa or 10uM Tryphostin for 2 hours prior to treatment with 200nM EGF. Cell were then harvested an hour later for analysis of EGFR by western blot. [file 1471-2407-12-368-S8.pdf]

ADDITIONAL FILE 8.

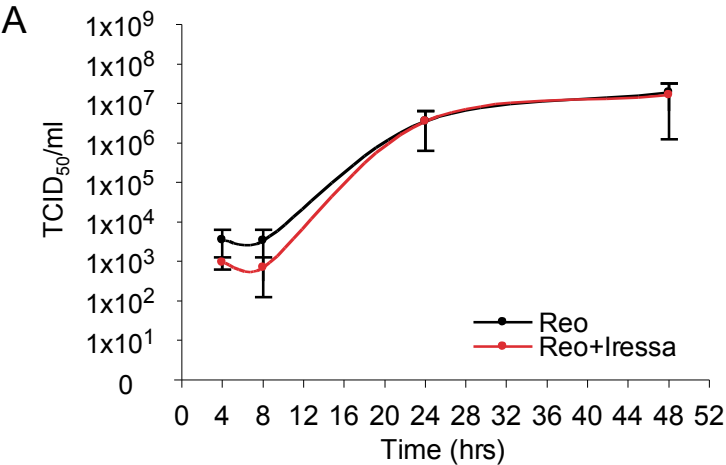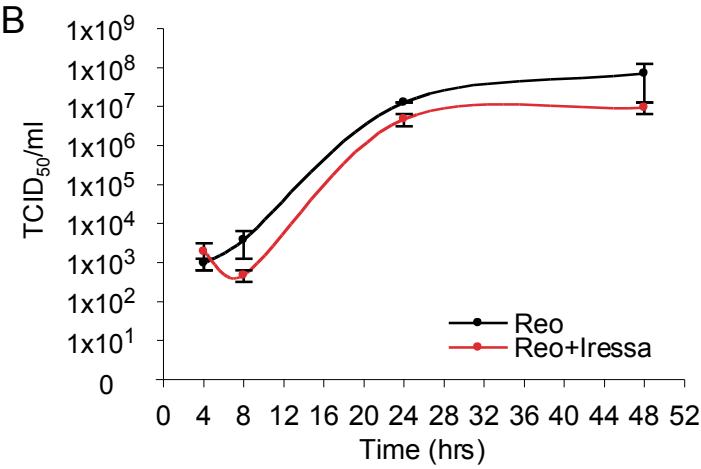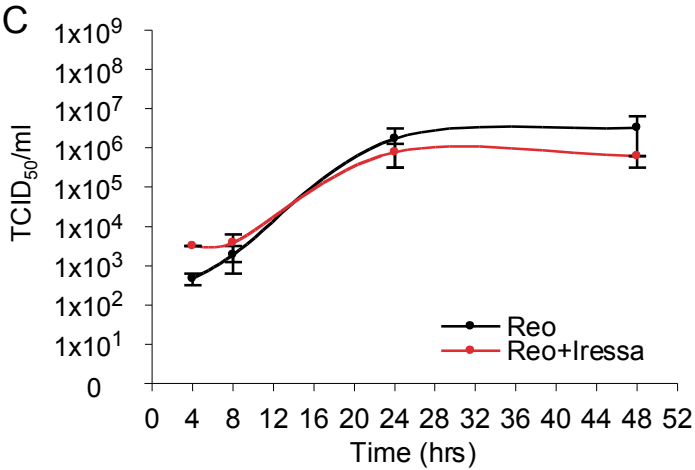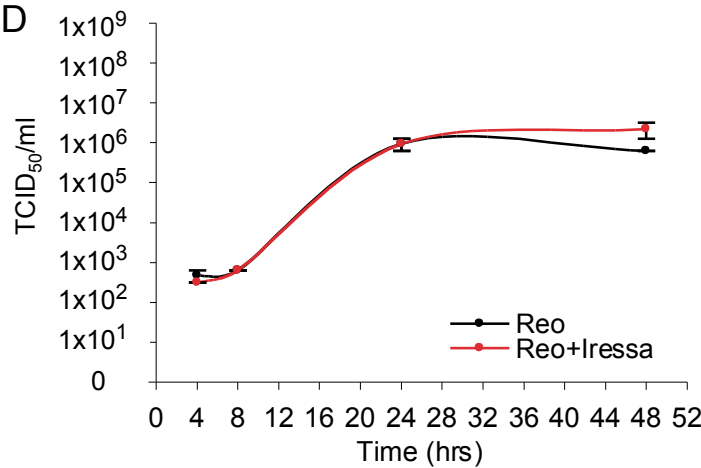

Supplement: Additional file 9 — Reovirus grows at the same rate in EGFR inhibited SCCHN cells as in untreated cells. (A) Cal27, (B) SIHN 5B, (C) HN3 and (D) HN5, were treated overnight with 1 μM Iressa then infected with reovirus (MOI 10) using viral stocks at 1.2×10^10^ TCID50/ml. Iressa was replaced 2 hrs post infection. Cells and supernatants were harvested at the times indicated for TCID50 titration on L929 cells. Means are from at least 2 independent experiments and error bars represent SEMs. [file 1471-2407-12-368-S9.pdf]

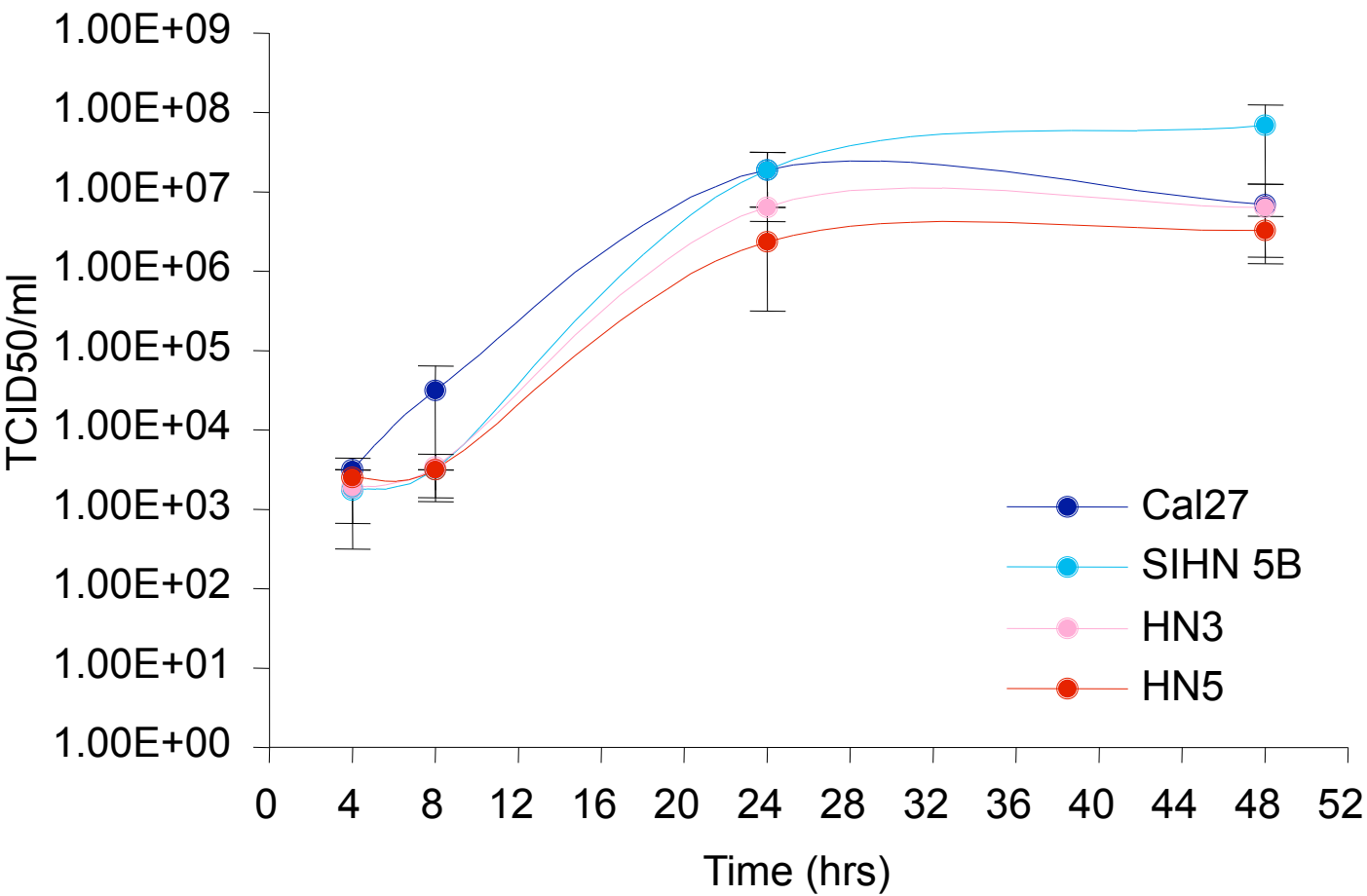

Supplement: Additional file 10 — Reovirus grows at the same rate in reovirus sensitive or resistant cells. Cal27, SIHN 5B HN3 and HN5 infected with reovirus (MOI 10) using viral stocks at 1.9×10^9^TCID50/ml. Cells and supernatants were harvested at the times indicated for TCID50 titration on L929 cells. Means are from at least 2 independent experiments and error bars represent SEMs. [file 1471-2407-12-368-S10.pdf]

ADDITIONAL FILE 10.

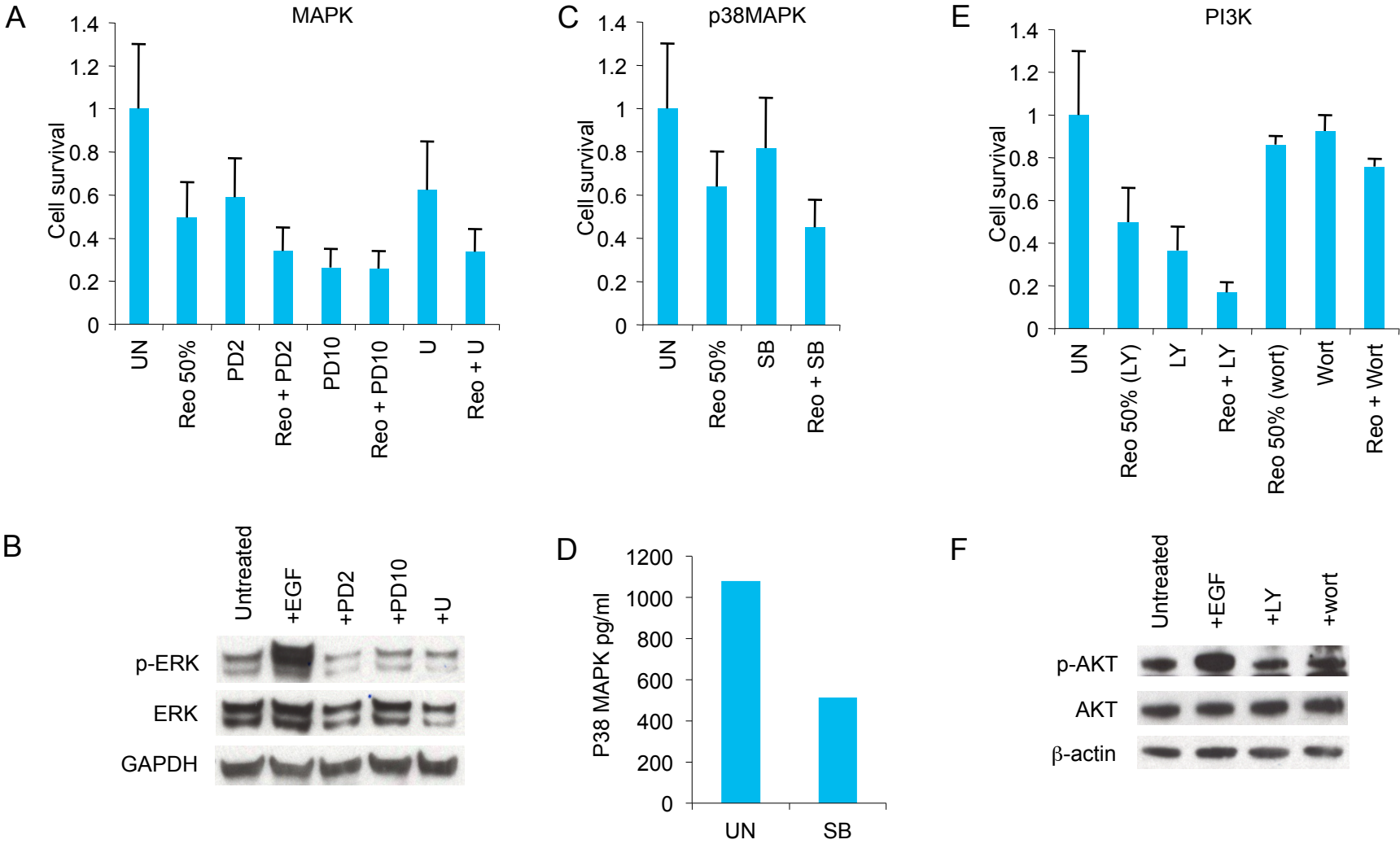

Supplement: Additional file 11 — MEK, PI3-K or p38MAPK inhibition does not affect reovirus cytotoxicity in SIHN-5B cells. Cells were inhibited for 2 hrs with 2 μM (PD2) or 10 μM (PD10) PD184352, 10 μM U0126 (U), 10 μM SB202190 (SB), 10 μM LY294003 (LY) or 1 μM wortmannin (wort). Monolayers were then either lysed, resolved on 8% Precise Protein Gels (MAPK) or 10% NuPage Novex Bis Tris gels (PI3-K) and probed for total ERK1/2, phosho-Thr202 ERK1/2, total AKT, phospho-Ser473 AKT and GAPDH or β-actin as loading controls, or infected with reovirus at 1.2×10^10^ TCID50/ml (PD, SB and LY) or 7.8×10^8^ TCID50/ml (wort) and assayed for cell survival by MTT. p38MAPK target knock-down was confirmed by ELISA. Reovirus was diluted at 1:6000 for 50% cell kill. A, B. MAPK inhibition. C, D. p38MAPK inhibition. E, F. PI3K inhibition. Means are calculated from at least 3 independent experiments and error bars represent SEMs. [file 1471-2407-12-368-S11.pdf]

ADDITIONAL FILE 11.

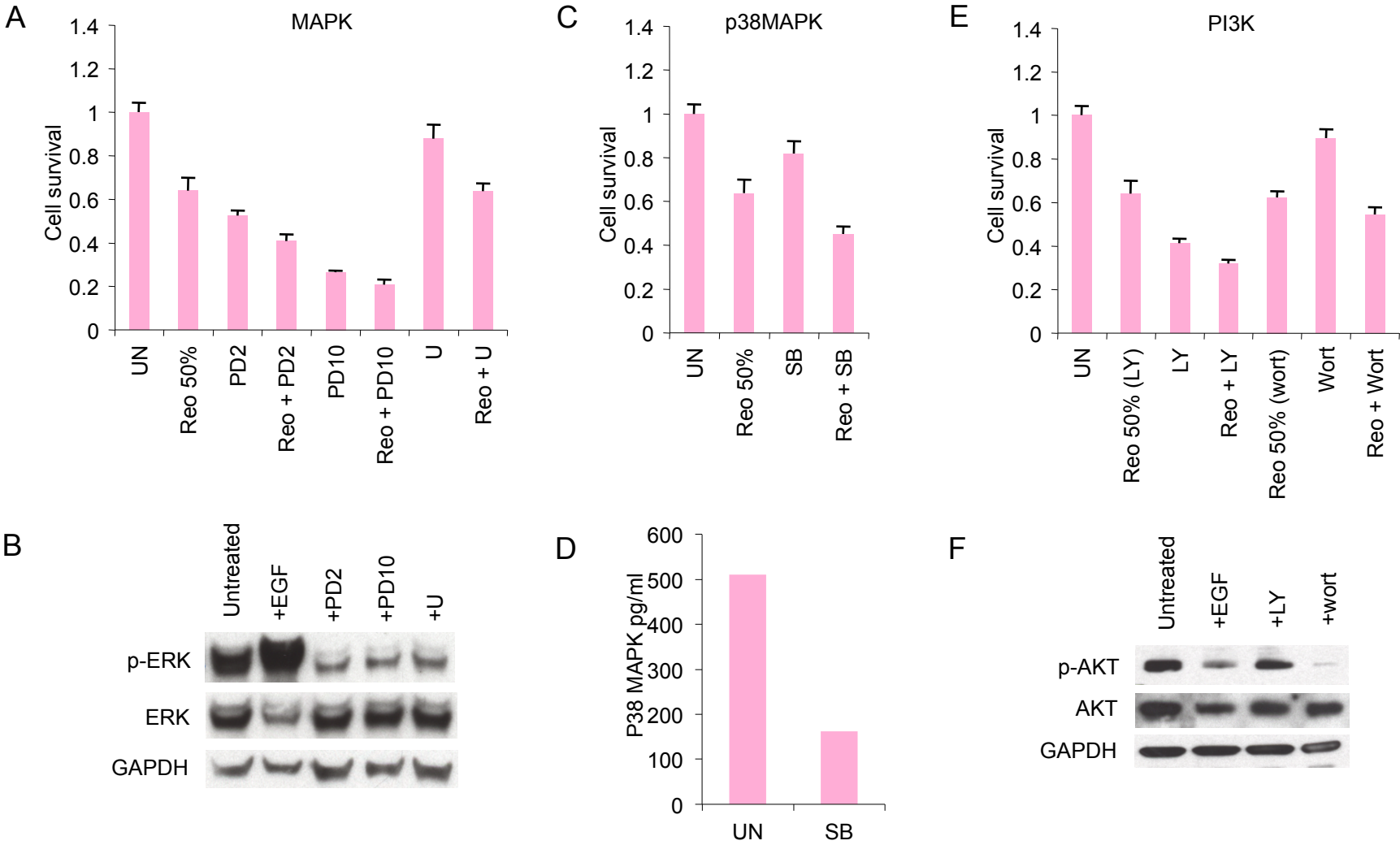

Supplement: Additional file 12 — MEK, PI3-K or p38MAPK inhibition does not affect reovirus cytotoxicity in HN3 cells. Cells were inhibited for 2 hrs with 2 μM (PD2) or 10 μM (PD10) PD184352, 10 μM U0126 (U), 10 μM SB202190 (SB), 10 μM LY294003 (LY) or 1 μM wortmannin (wort). Monolayers were then either lysed, resolved on 8% Precise Protein Gels (MAPK) or 10% NuPage Novex Bis Tris gels (PI3-K) and probed for total ERK1/2, phosho-Thr202 ERK1/2, total AKT, phospho-Ser473 AKT and GAPDH or β-actin as loading controls, or infected with reovirus at 1.2×10^10^ TCID50/ml (PD, SB and LY) or 7.8×10^8^ TCID50/ml (wort) and assayed for cell survival by MTT at 96 hours post-infection. p38MAPK target knock-down was confirmed by ELISA. Reovirus was diluted at 1:2000 for 50% cell kill. A, B. MAPK inhibition. C, D. p38MAPK inhibition. E, F. PI3K inhibition. Means are calculated from at least 3 independent experiments and error bars represent SEMs. [file 1471-2407-12-368-S12.pdf]

ADDITIONAL FILE 12.

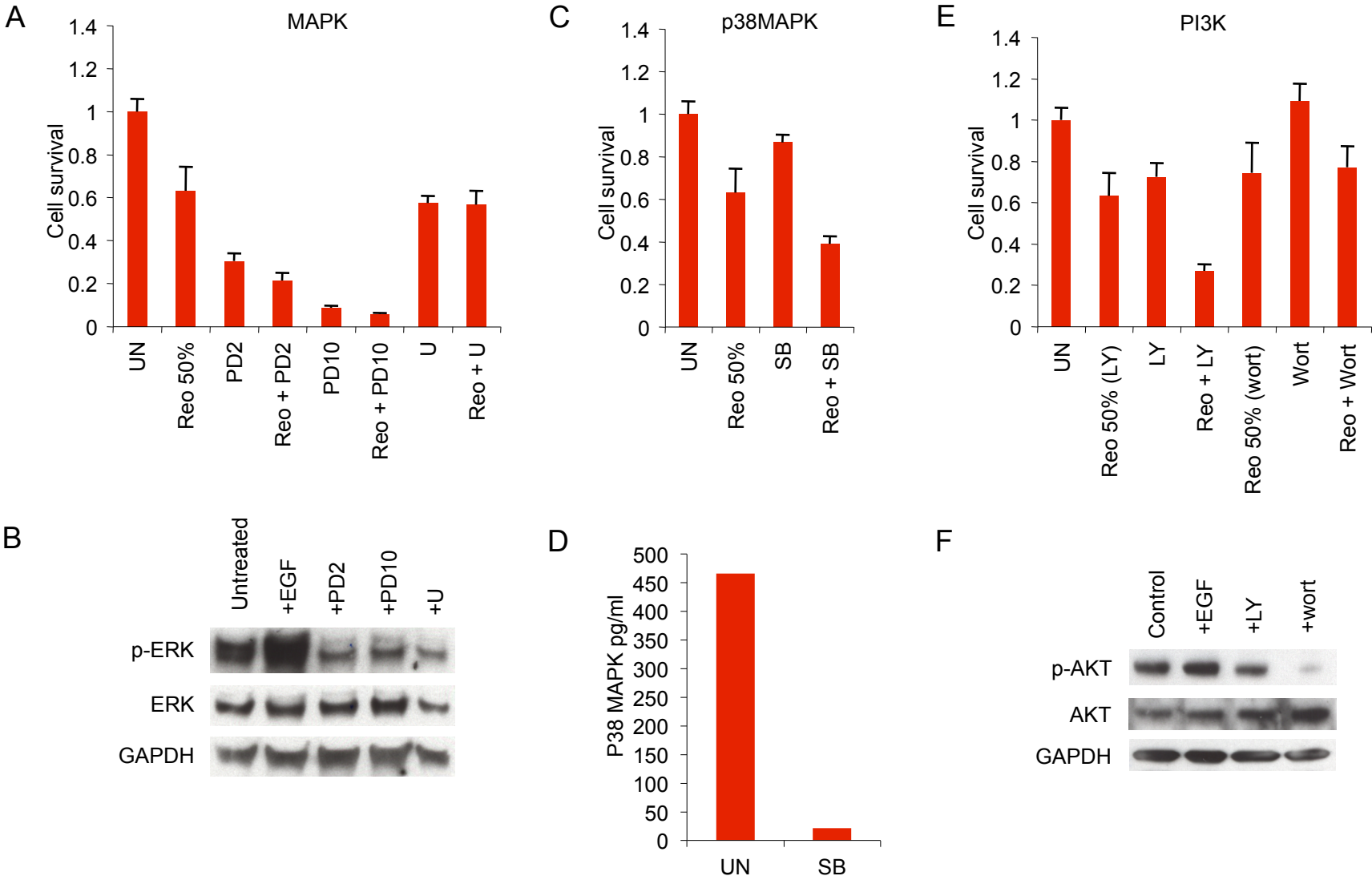

Supplement: Additional file 13 — MEK, PI3-K or p38MAPK inhibition does not affect reovirus cytotoxicity in HN5 cells. Cells were inhibited for 2 hrs with 2 μM (PD2) or 10 μM (PD10) PD184352, 10 μM U0126 (U), 10 μM SB202190 (SB), 10 μM LY294003 (LY) or 1 μM wortmannin (wort). Monolayers were then either lysed, resolved on 8% Precise Protein Gels (MAPK) or 10% NuPage Novex Bis Tris gels (PI3-K) and probed for total ERK1/2, phosho-Thr202 ERK1/2, total AKT, phospho-Ser473 AKT and GAPDH or β-actin as loading controls, or infected with reovirus at 1.2×10^10^ TCID50/ml (PD, SB and LY) or 7.8×10^8^ TCID50/ml (wort) and assayed for cell survival by MTT at 96 hours post-infection. p38MAPK target knock-down was confirmed by ELISA. Reovirus was diluted at 1:100 for 50% cell kill. A, B. MAPK inhibition. C, D. p38MAPK inhibition. E, F. PI3K inhibition. Means are calculated from at least 3 independent experiments and error bars represent SEMs. [file 1471-2407-12-368-S13.pdf]

A.

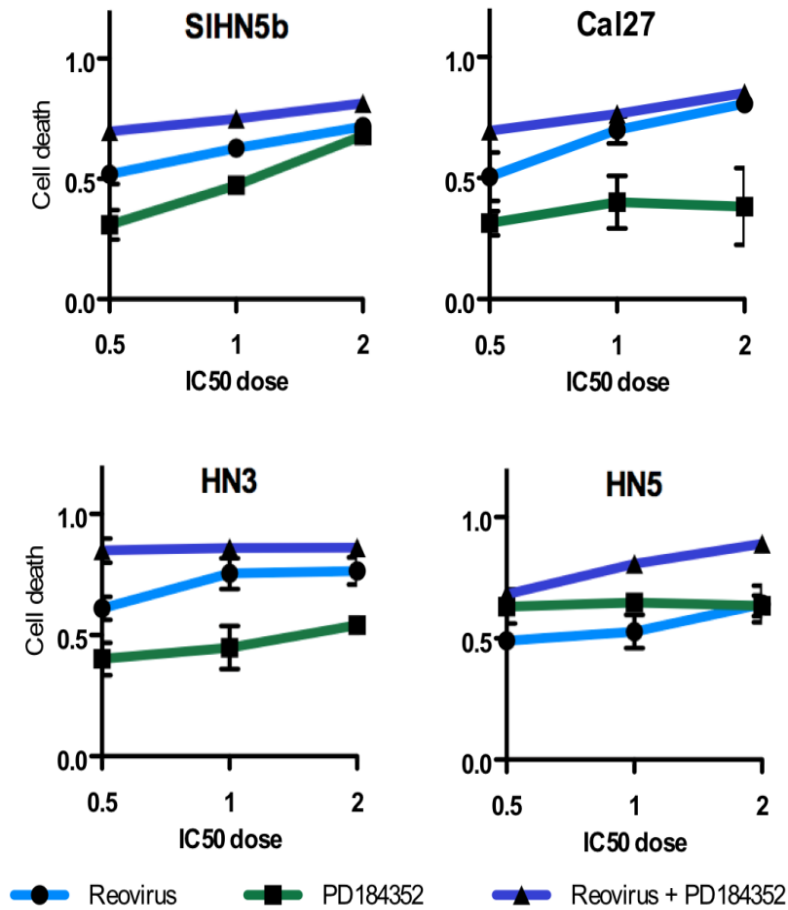

B.

|        | 2x    | 1x    | 0.5x  |
|--------|-------|-------|-------|
| Cal27  | 0.773 | 0.72  | 0.53  |
| HN3    | 0.478 | 0.242 | 0.14  |
| HN5    | 0.05  | 0.087 | 0.166 |
| SIHN5b | 0.56  | 0.524 | 0.412 |

|        |       |       |      |
|--------|-------|-------|------|
| Cal27  | ++    | ++    | +++  |
| HN3    | +++   | ++++  | ++++ |
| HN5    | +++++ | +++++ | ++++ |
| SIHN5b | +++   | +++   | +++  |

Synergy

Additive

Antagonism

Supplement: Additional file 14 — Reovirus in combination with PD184352 is synergistic. Cal27, HN3, HN5 SIHN5b were plated at 5×10^3^/ 96 well dish and treated the following day with 200 μl of 2x, 1x, 0.5x calculated IC50 doses of reovirus, PD184352, or the agents in combination. Cells were analysed for cell survival 96 hours later by MTT assay at 96 hours post-infection. Data are derived from 2 independent experiments ± SEM. A. Interaction between the treatment combinations was assessed using the method of Chou and Talalay [34]. Combination index (CI) values were calculated where CI <0.9 is classed as synergy, 0.9-1.1 is additive and >1.1 is antagonistic. B. Representative CI values for the combination of reovirus with PD184352 for Cal27, HN3, HN5 and SIHN5b. [file 1471-2407-12-368-S14.pdf]

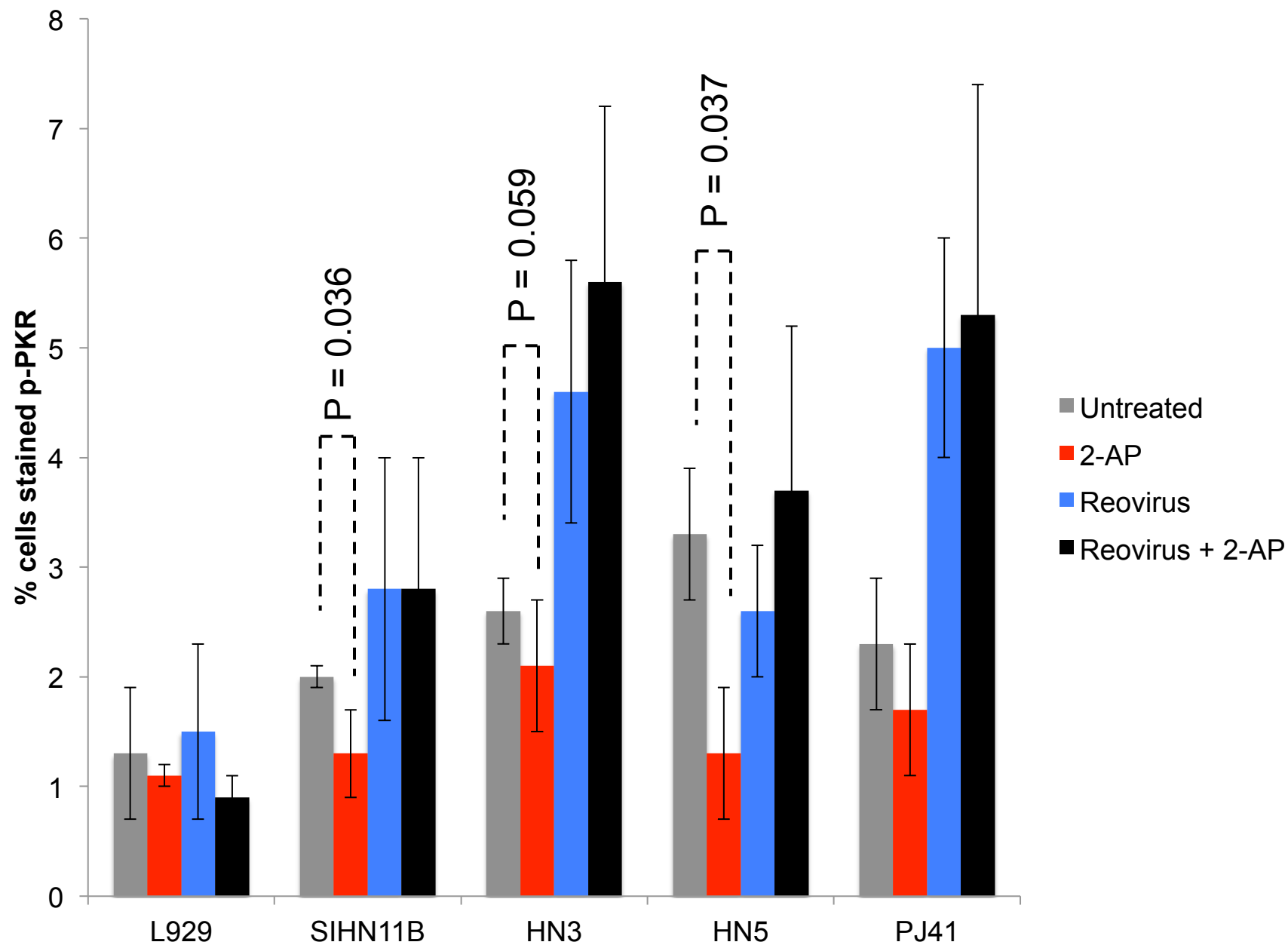

Supplement: Additional file 15 — L929, SIHN11b, HN3, HN5 and PJ41 cells were seeded at 1 × 10^6^in 10 cm dishes, treated the following day with reovirus and were fixed with 10% formalin at room temperature for 4–8 hours then harvested and centrifuged at 1200 rpm for 5 minutes. The remaining pellet was resuspended in sterile DEPC water before analysis by immunocytochemistry staining for p-PKR (by G. Nuovo). (PDF 137 kb) [file 1471-2407-12-368-S15.pdf]

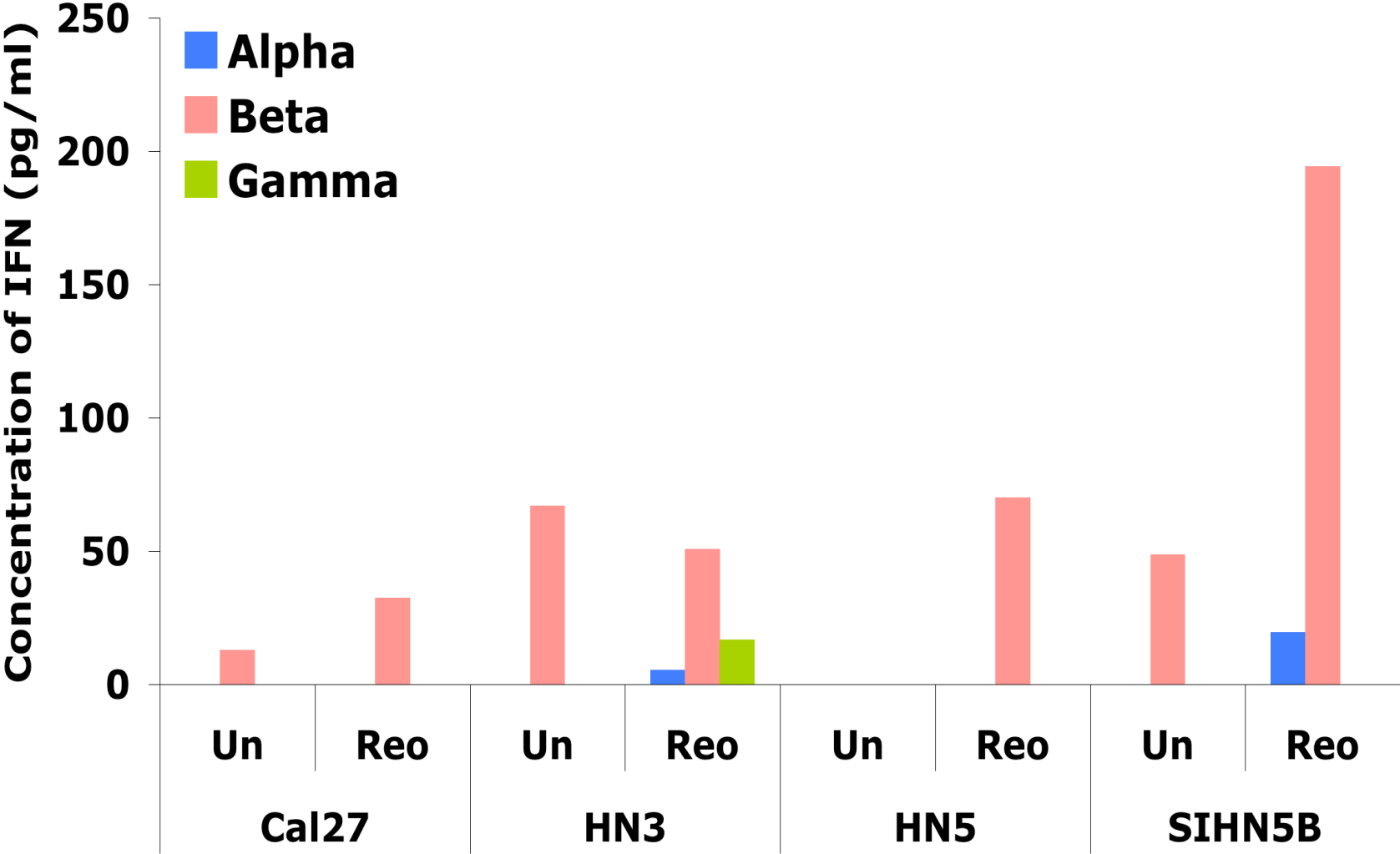

Supplement: Additional file 16 — Interferon response does not predict reovirus sensitivity. Cal27, HN3, HN5 SIHN5b were plated at 1×106/10 cm dish and the following day treated with reovirus at an MOI of 5 (reo), or left untreated (Un). Cells were incubated for 24 hours and the the supernatants were harvested, spun down at 1200 rpm for 4 minutes to remove cells and debris, and the supernatant transferred to a fresh tube and stored at −20°C. Samples were analysed in triplicate for interferons by ELISA. [file 1471-2407-12-368-S16.pdf]
